# Representation of color, form, and their conjunction across the human ventral visual pathway

**DOI:** 10.1016/j.neuroimage.2022.118941

**Published:** 2022-02-02

**Authors:** JohnMark Taylor, Yaoda Xu

**Affiliations:** a Visual Inference Laboratory, Zuckerman Institute, Columbia University, United States; b Department of Psychology, Yale University, United States

**Keywords:** Color, Ventral stream, Feature binding, Early visual cortex

## Abstract

Despite decades of research, our understanding of the relationship between color and form processing in the primate ventral visual pathway remains incomplete. Using fMRI multivoxel pattern analysis, we examined coding of color and form, using a simple form feature (orientation) and a mid-level form feature (curvature), in human ventral visual processing regions. We found that both color and form could be decoded from activity in early visual areas V1 to V4, as well as in the posterior color-selective region and shape-selective regions in ventral and lateral occipitotemporal cortex defined based on their univariate selectivity to color or shape, respectively (the central color region only showed color but not form decoding). Meanwhile, decoding biases towards one feature or the other existed in the color- and shape-selective regions, consistent with their univariate feature selectivity reported in past studies. Additional extensive analyses show that while all these regions contain independent (linearly additive) coding for both features, several early visual regions also encode the conjunction of color and the simple, but not the complex, form feature in a nonlinear, interactive manner. Taken together, the results show that color and form are encoded in a biased distributed and largely independent manner across ventral visual regions in the human brain.

## Introduction

Research over the past several decades has provided us with a wealth of knowledge regarding the representation of color and form information in the primate brain. Both color and form information have been shown to be represented and transformed across multiple levels of processing, with the relevant neural processes spanning the entire visual processing hierarchy, from the retina to higher-level ventral stream regions. Notably, human fMRI studies have identified form-processing regions in lateral and ventral occipito-temporal cortex (OTC) (Malach et al., 1995; Grill-Spector et al., 1998; Kourtzi and Kanwisher, 2001; Orban et al., 2004), and both monkey neurophysiology and human fMRI studies have reported color-processing regions in ventral OTC (Hadjikhani et al., 1998; Brewer et al., 2005; Conway et al., 2007; Lafer-Sousa and Conway, 2013; Lafer-Sousa et al., 2016; Chang et al., 2017; Conway, 2018). Despite these advances, past studies tended to examine a single feature in isolated brain regions with a range of different methods or stimuli, making it difficult to construct an overarching view of how color and form are coded relative to each other within a brain region and across different regions along the primate ventral processing pathway.

In the present study, we address these limitations by comprehensively probing visual processing regions along the ventral visual pathway with the same stimuli to document the extent to which color and form are encoded in overlapping versus independent brain regions, compare the magnitude of color and form decoding for the regions that encode both (allowing us to test whether regions that have shown univariate selectivity for a given feature exhibit a corresponding multivariate feature preference), and determine whether regions with information about both features encode it in an additive versus interactive manner. We draw on a paradigm developed by Seymour et al. (2010) that uses fMRI and multi-voxel pattern analysis (MVPA) to examine color and form coding, both replicating their results and extending them in two ways. First, in addition to the early visual areas examined in their study, we examined higher-level ventral stream regions exhibiting univariate selectivity to either color or shape information. Second, in addition to examining the coding of color and orientation (a low-level form feature) as in their study, we documented the coding of color and a mid-level form feature, curvature, across the early visual and ventral visual regions. Together, our approach provides an updated documentation of the representation of color, form, and their conjunction across the human ventral visual pathway.

### Color and form processing across the visual hierarchy

Past work has demonstrated that both color and form information is successively transformed across a series of processing stages, spanning from early visual cortex to anterior temporal lobe regions. Early visual areas V1 to V4 have been shown to contain both color and shape information, with some degree of mesoscale segregation of neurons tuned to each of these two features (e.g., Livingstone and Hubel, 1988; Gegenfurter et al., 1996; Conway, 2001; Johnson et al., 2001; Ts’o et al., 2001; Brewer et al., 2005; Conway et al., 2007; Brouwer and Heeger, 2009; Conway et al., 2010; Seymour et al., 2010; Shapley and Hawken, 2011).

For higher ventral regions beyond V4, coding for color and form exhibit more anatomical separation. Specifically, macaque inferotemporal cortex (IT) contains neurons tuned to high-level shape features (e.g., Tanaka, 1996; DiCarlo et al., 2012; Lehky and Tanaka, 2016; Bao et al., 2020), and arguably homologous regions in the human lateral and ventral OTC exhibit higher fMRI responses to coherent shapes than scrambled stimuli (Malach et al., 1995; Grill-Spector et al., 1998; Kourtzi and Kanwisher, 2001; Orban et al., 2004). Critically, damage to these cortical regions can result in loss of form perception, with spared color perception (Benson and Greenberg, 1969; Goodale and Milner, 2004). Analogously, a series of posterior, central and anterior color-selective regions in ventral visual cortex have been shown to exhibit color-tuning in the macaque, and show higher fMRI responses to colored than greyscale stimuli in the human brain (Hadjikhani et al., 1998; Brewer et al., 2005; Conway et al., 2007; Lafer-Sousa and Conway, 2013; Lafer-Sousa et al., 2016; Chang et al., 2017; Conway et al., 2018). Damage to these color regions has been linked to deficits in color processing with largely spared form processing (Siuda-Krzywicka and Bartolomeo, 2020; Bouvier and Engel, 2006).

The existence of regions reliably showing univariate selectivity to color and form, along with the lesion data, is consistent with the view that different features are encoded by anatomically distinct neural populations in high-level vision. However, color and form information may be encoded in distributed, fine-grained activation patterns that univariate mean-activation methods cannot detect (e.g., Haxby et al., 2001). Indeed, macaque IT and color regions contain both color and form information (Komatsu and Ideura, 1993; McMahon and Olson, 2009; Chang et al., 2017; Rosenthal et al., 2018; Duyck et al., 2021) and the human shape-selective region in lateral occipital cortex has been found to contain color information using fMRI multivoxel pattern analysis (Bannert and Bartels, 2013, 2018).

How should we reconcile the existence of regions showing univariate selectivity for color or form with the evidence suggesting that tuning for these features might be broadly distributed throughout the ventral visual pathway? A primary goal of the present study is to systematically document the multivariate coding of color and form throughout the human ventral visual processing pathway, compare how the relative coding strength of these two types of features may vary across brain regions, and determine whether regions exhibiting univariate feature selectivity as reported in previous work would show a similar bias in multivariate feature decoding.

### Independent or interactive coding of color and form?

The mesoscale segregation of neurons specialized for processing color and form features in early visual areas, the existence of distinct higher-level visual regions showing univariate selectivity to color or form, and the behavioral deficits associated with damage to these regions are consistent with independent coding of color and form in the primate brain. Available psychophysical evidence also supports this view, such as from visual search and illusory conjunction effects (e.g., Treisman and Gelade, 1980; Treisman and Schmidt, 1982), leading Treisman and Gelade (1980) to posit *feature integration theory*: different visual features are initially encoded on their own distinct feature maps, and focused attention then spatially links the different features associated with the same object to encode conjunctions of features.

Meanwhile, various behavioral studies have shown that color and form may be automatically encoded in a conjoined and interactive format without requiring a separate, laborious attention-driven binding step (e.g., Stromeyer, 1969; Victor et al., 1989; Cavanagh, 1991; Heywood et al., 1991; Barbur et al., 1994, 1998; Holcombe and Cavanagh, 2001; Mandelli and Kiper, 2005). At the level of neural coding, non-additive (interactive) feature coding has been found in human early visual areas (Engel, 2005; Seymour et al., 2010; see more details of the latter study below) and macaque V4 and color regions (Bushnell and Pasupathy, 2012, and Chang et al., 2017). It is largely absent in IT, and has not been explicitly tested in V1 and V2 in macaques (Friedman et al., 2003; McMahon and Olson, 2009). Notably, interactive feature coding in the human brain has thus far only been tested for simple form features, such as orientation, leaving it unknown whether this processing format is also used for the conjunction of color with more complex form features.

A second goal of the present study is thus to examine the prevalence of non-additive color and form coding in the ventral visual pathway by testing whether it is present for both the conjunction of color and simple form features and that of color and more complex form features, and determining whether it can be found in lower as well as higher ventral regions in the human brain. Due to the “combinatorial explosion” involved in directly encoding every possible combination of color and form features, it is possible that interactive coding may only be used for some form features but not others, making it important to determine how broadly it applies.

### Present study

**Fig. 1.**
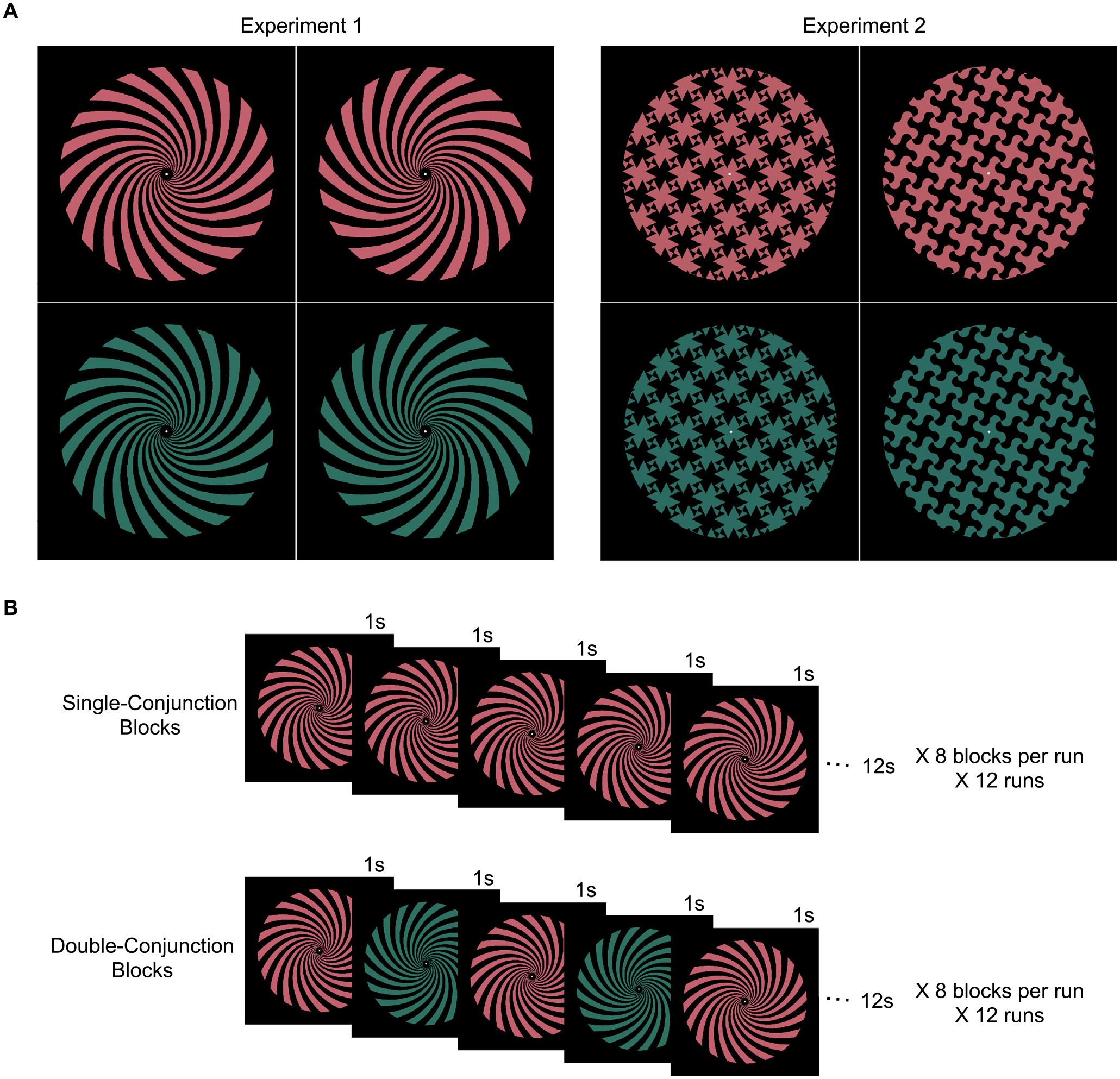
Stimuli and experimental design. **A.** In Experiment 1 (left), logarithmic spiral stimuli (adapted from Seymour et al., 2010) were shown that could be oriented clockwise or counterclockwise, and colored red or green. These spirals have the property that their arms are a fixed angle from the radius at all points, ensuring that gross radial biases in cortical retinotopic maps could not drive decoder performance. In Experiment 2 (right), spiky and curvy tessellation stimuli were used, with the same colors as Experiment 1. The stimuli alternated phase once per second, such that black shapes within the circular aperture became colored, and vice versa. **B.** The two kinds of blocks present in both experiments. Stimuli were either presented in single-conjunction blocks, where a single stimulus type (e.g., Red-CW spiral) was presented for the entire block with its phase alternating once per second, or in double-conjunction blocks, where stimuli varying with respect to both features alternated once per second within a block. Thus, in Experiment 1, the two kinds of double-conjunction block were Red-CW/Green-CCW and Red-CCW/Green-CW; in Experiment 2, the two kinds of double-conjunction block were Red-Spiky/Green-Pinwheel and Red-Pinwheel/Green-Spiky.

To answer the outstanding questions raised above, we replicated and extended a previous human fMRI MVPA study by Seymour et al. (2010) that examined color and orientation coding in early visual areas. In this study, spiral stimuli were shown that were either clockwise or counterclockwise, and either red or green (see Fig. 1 for an illustration). In the *single conjunction condition*, spiral stimuli for each orientation and color combination were shown in different blocks of trials, with the phase of each spiral alternating over the course of the block to ensure that any form decoding was not a confound from differing retinotopic footprints of the stimuli. fMRI decoding revealed the presence of both color and orientation information in V1, V2, V3, and V4. In the *double conjunction condition*, *pairs* of stimuli with both features differing (e.g., either Red-Clockwise and Green-Counterclockwise, or Red-Counterclockwise and Green-Clockwise) were shown alternating throughout a block of trials, such that the two kinds of block had the same individual features, but differed in how they were conjoined. fMRI decoding revealed interactive coding of color and orientation throughout V1 to V4. These regions thus appeared to encode not just color and orientation, but also how they were combined.

While Seymour et al. (2010) was elegantly designed and theoretically informative, it was limited to the coding of color and orientation in early visual areas. To address the two main questions we raised earlier, here we extended Seymour et al. (2010) in two important ways: first, we examine not only early visual regions but also higher-level ventral stream regions defined based on their univariate selectivity to either color or form, and second, we examine color and form coding not just for a simple form feature (orientation), but also a mid-level form feature, curvature. Our study additionally allowed us to replicate the interactive coding for color/orientation conjunctions in early visual areas as reported by Seymour et al. (2010) and test whether such a coding scheme is specific to simple form features in early visual areas, or is a broader motif of color-form processing in human ventral cortex. As an additional extension of their study, we devised a new analysis technique, *pattern difference MVPA*, that we used as a further method to probe for interactive color-form tuning; this method can also be used to look for subtle interaction effects in fMRI paradigms beyond the present study.

We found that color and form could be decoded from activity in early visual areas V1 to V4, as well as in the posterior color-selective region and shape-selective regions in ventral and lateral occipitotemporal cortex defined based on their univariate selectivity to color or shape, respectively (the central color region only showed color but not form decoding). Meanwhile, decoding bias towards one feature or the other existed in the color- and shape-selective regions, largely consistent with their univariate feature selectivity reported in past studies. While all regions encoding both color and shape contained independent (linearly additive) coding of the two features, several analyses found evidence that early visual cortex additionally contains a tuning component that encodes the conjunction of color and the simple, but not the complex, form feature in a nonlinear, interactive manner. Taken together, the results show that color and form are encoded in a biased distributed and largely independent manner across ventral visual regions in the human brain.

## Materials and methods

### Participants

Experiment 1 included 12 healthy, right-handed adults (7 females, between 25 and 34 years old, average age 30.6 years old) with normal color vision and normal or corrected to normal visual acuity. Experiment 2 included 13 healthy adults (7 females, between 25 and 34 years old, average age 28.7 years old). Four participants partook in both experiments. Participants were members of the Harvard community with prior scanning experience. All participants gave informed consent prior to the experiments and received payment. The experiments were approved by the Committee on the Use of Human Subjects at Harvard University.

### Stimuli

#### Experiment 1: Colored spirals

Stimulus design and experimental design for Experiment 1 were largely adapted from Seymour et al. (2010), with identical stimuli and tasks but some differences in the number and timing of the blocks. Participants viewed colored spiral stimuli that varied by color—red or green—and orientation—clockwise (CW) or counterclockwise (CCW)—resulting in four different kinds of spirals (Fig. 1A). Spirals were presented on a black background.

The spirals used were *logarithmic spirals*, defined by the formula *r*=ae^b*θ*^, which have the property that the angle between the radius of the spiral and an arm of the spiral at any point is fixed, in this case at 45°. This property ensures that there is a constant relationship between the location of an edge of a spiral arm in visual space and the radial component of its angle, as would not be the case if oriented gratings were used (for example, a horizontal oriented grating would have a maximal radial component along the horizontal midline, and minimal radial component along the vertical midline). This constraint accounts for the known *radial bias* in early visual cortex, in which radial orientations are preferentially represented in early visual topographic maps (e.g., zones of cortex corresponding to the top of the visual field have an over-representation of vertically oriented angles), ensuring that successful decoding of orientation could not simply be due to activation of different sub-regions of topographic maps (Sasaki et al., 2006; Mannion et al., 2009; Seymour et al., 2010). Stimuli were generated by first drawing 40 spiral lines at evenly spaced angles from the origin according to the above formula and filling in alternating regions of the spiral with the stimulus color and the background color, black, resulting in 20 spiral arms. The spiral subtended a circular region covering 9.7° of visual angle, with an internal aperture in the middle, within which a white fixation dot was displayed. As mentioned earlier, the spiral arms could be oriented either clockwise or counterclockwise. Additionally, depending on which of the spiral arms were colored and which were black, each spiral could be presented in one of two phases.

The exact spiral colors used in the experiment were generated using the following procedure. To generate initially isoluminant shades of red and green, each participant performed a flicker-adjustment procedure inside the scanner (Kaiser, 1991), in which a flickering checkerboard with the two colors being adjusted flashed at 30 hz, and participants adjusted the colors until the flickering sensation was minimal. Specifically, the two colors had RGB values of the form red-hue = [178, 178 - X, 89] and green-hue = [0, X, 89], where participants adjusted the “X” parameter until isoluminance was achieved. This procedure guarantees that the two colors are isoluminant and sum to neutral gray, thereby equally stimulating all chromatic channels. Participants performed ten trials of this procedure, and the average “X” value was used to produce the initial colors. However, since this procedure might theoretically have some associated imprecision, each color was presented at either +/−10% of its initially calibrated luminance value on any given run of the experiment, where the number of high-luminance and low-luminance runs was balanced across the red and green colors. This manipulation ensures that any residual between-hue luminance differences will be far smaller than the within-hue luminance differences, reducing the likelihood that luminance, rather than hue, could drive MVPA classification during analysis. The luminance adjustment procedures were identical to those of Seymour et al. (2010), with the minor difference that their study varied the luminance settings of a given color within a run, whereas we varied it between runs.

#### Experiment 2: Colored tessellation patterns

For this experiment, we constructed two different tessellation stimuli, consisting either of a curvy or a spiky pattern within a circular aperture (Fig. 1A). These stimuli were deliberately designed so as not to resemble any real-world entities, and we decided upon a curvy versus spiky contrast because curvature is a salient mid-level visual feature, in contrast with orientation, which can be considered a lower-level visual feature (Gallant et al., 1993; Srihasam et al., 2014; Yue et al., 2014). The “phase” of the tessellation stimuli could also vary, based on whether a given region of the stimulus was currently colored or black. Exactly the same procedure as Experiment 1 was used to calibrate the colors of the two stimuli, and the stimuli subtended the same visual angle (9.7°) as in Experiment 1.

### Procedure

#### Experiment 1: Colored spirals

Participants viewed 12 s blocks of the stimuli and had to detect a 30% luminance increment or decrement using a button press (index finger for increase, middle finger for decrease). On any given block, two 500 ms luminance changes were presented, one in the first half and one in the second half of the block, and never in the first or last two stimuli of the block. The number and timing of the increments and decrements within the blocks was balanced across the whole experiment, and across all stimulus conditions described below. There were 9 s fixation blocks between the stimulus blocks and at the end of the run, with a 12 s fixation block at the beginning of the run. This allowed us to better separate fMRI responses from adjacent blocks (note that Seymour et al., 2010 included no fixation blocks between stimulus blocks in their design).

The experiment included two kinds of runs (Fig. 1B). In the *single-conjunction runs*, only a single kind of spiral (RedCW, RedCCW, GreenCW, or GreenCCW) was presented for a given block, with its phase alternating once per second (for a total of 12 phase alternations per block), with no blank period between phase alternations within a block (i.e., the alternations between successive phases were instantaneous). This phase alternation ensures that all conditions were equated in their retinotopic footprint over the course of each block, removing this as a possible confound in form decoding. Since two starting phases were possible, each of the four spiral types could begin on either starting phase, resulting in 8 different block types for these runs. Each run contained one instance of each of the 8 types of block, totaling 180 s per run. Participants completed 12 such runs, thus viewing a total of 24 blocks of each of the four spiral types over the whole session. To ensure that block types were roughly matched in terms of their placement within the runs and how frequently they appeared next to other block types, a random balanced Latin square procedure was used to generate the block order for each subject; specifically, two random 8 × 8 balanced Latin squares were generated, the second square was truncated to 4 × 8, and the two squares were concatenated, giving the block order for 12 runs of 8 blocks each.

In the *double-conjunction runs*, there were two block conditions: a block could either alternate between RedCW and GreenCCW, or between RedCCW and GreenCW, with the phase of each spiral type alternating at each presentation; for instance, an example block would progress through the sequence RedCW-Phase1, GreenCCW-Phase2, RedCW-Phase2, GreenCCW-Phase1, and then repeat. Since each block condition could begin on either one of the two spirals in either one of the two phases, there were therefore four different block types for each block condition. Due to how the spirals were constructed and how the stimuli alternated phase within each block type, varying the starting stimulus in this manner ensured that the two block conditions were matched in how frequently each pixel took on values of red (25% of the time), green (25% of the time), and black (50% of the time) both over the course of any given block and at any given time point across the four block types within each block condition. This ensured that pixel-level information could not drive decoding during the MVPA analysis. The stimulus timing, number of blocks, counterbalancing method, and task for these runs was otherwise identical to that of the single-conjunction runs. Participants completed 12 double-conjunction runs, and thus viewed each kind of double conjunction block 48 times. The single-conjunction runs and double-conjunction runs alternated in sets of three (e.g., three double-conjunction runs, then three single-conjunction runs), with the type of the initial run set counterbalanced across participants. Note that while Seymour et al. (2010) interleaved single-conjunction blocks and double-conjunction blocks within the same run, we separated them into different runs. This allowed us to form two completely independent datasets to more rigorously validate results showing interactive coding of color and form.

#### Experiment 2: Colored tessellation patterns

Exactly the same task and experimental design were used in Experiment 2 as in Experiment 1, with only the stimuli varying. Due to how the tessellation stimuli were constructed and the manner in which they alternated phase within the double conjunction blocks, they shared with the spirals the property that each pixel was matched in its frequency of taking on values of red, green, and black both over the course of the block, and at corresponding timepoints for the two block conditions, across the four block types within each block condition.

#### Localizer experiments

As regions of interest in both experiments, we included retinotopically-defined regions V1, V2, V3, and V4 in early visual cortex, and functionally-defined shape and color regions in occipitotemporal visual cortex.

To localize topographic visual field maps, we followed standard retinotopic mapping techniques (Sereno et al., 1995). A 72° polar angle wedge swept either clockwise or counterclockwise (alternating each run) across the entire screen, with a sweeping period of 36.4 s and 10 cycles per run. The entire display subtended 23.4 × 17.6° of visual angle. The wedge contained a colored checkerboard pattern that flashed at 4 Hz. Participants were asked to detect a dimming in the polar angle wedge. Each participant completed 4–6 runs, each lasting 364 s.

We localized two shape regions in lateral occipitotemporal (LOT) and ventral occipitotemporal (VOT) cortex, following the procedure described by Kourtzi and Kanwisher (2001), and subsequently used in several of our own lab’s studies (Vaziri-Pashkam and Xu, 2017; Vaziri-Pashkam et al., 2019). LOT and VOT approximately correspond to the locations of LO and pFs (Malach et al., 1995; Grill-Spector et al., al., 1998; Kourtzi and Kanwisher, 2001) but extend further into the temporal cortex in order to include as many form-selective voxels as possible in occipitotemporal regions. Specifically, in a separate scanning session from the main experiment (usually the same one as the retinotopic mapping session), participants viewed black-and-white pictures of faces, places, common objects, arrays of four objects, phase-scrambled noise, and white noise in a block design paradigm, and responded with a button press whenever the stimulus underwent a slight spatial jitter, which occurred randomly twice per block. Each block contained 20 images from the same category, and each image was presented for 750 ms each, followed by a 50 ms blank display, totaling 16 s per block, with four blocks per stimulus category. Each run also contained a 12 s fixation block at the beginning, and an 8 s fixation block in the middle and end. Images subtended 9.5° of visual angle. Participants performed either two or three runs, each lasting 364 s.

We also localized a series of color-selective regions in ventral temporal cortex, using a procedure similar to Lafer-Sousa et al. (2016). Two runs of a color localizer were presented during the main scan session, one at the middle and one at the end of the session. In these runs, participants viewed 16 s blocks consisting of either colorful, highly saturated natural scene images selected from the online Places scene database (Zhou et al., 2018) or greyscale versions of these images. Participants responded when an image jittered back and forth, which occurred twice per block. Images subtended 9.5° of visual angle, and were each presented for 750 ms (50 ms blank period between stimulus presentations within a block). Each run contained 16 blocks, 8 for each of the two stimulus types, for a total run duration of 292 s including an initial 20 s fixation block, and an 8 s fixation block in the middle and the end of the run.

### MRI methods

MRI data were collected using a Siemens PRISMA 3T scanner, with a 32-channel receiver array headcoil. Participants lay on their backs inside the scanner and viewed the back-projected display through an angled mirror mounted inside the headcoil. The display was projected using an LCD projector at a refresh rate of 60 Hz and a spatial resolution of 1280×1024. An Apple Macbook Pro laptop was used to create the stimuli and collect the motor responses. Stimuli were created using Matlab and Psychtoolbox (Brainard 1997).

A high-resolution T1-weighted structural image (1.0 × 1.0 × 1.3 mm) was obtained from each participant for surface reconstruction. All Blood-oxygen-level-dependent (BOLD) data were collected via a *T*_2_*-weighted echo-planar imaging (EPI) pulse sequence that employed multiband RF pulses and Simultaneous Multi-Slice (SMS) acquisition. For the two main experiments, including the color localizer runs, 69 axial slices tilted 25° towards coronal from the AC-PC line (2 mm isotropic) were collected covering the whole brain (TR = 1.5 s, TE = 30 ms, flip angle = 75°, FOV = 208 m, matrix = 104×104, SMS factor = 5). For the retinotopic mapping and LOC localizer sessions, 64 axial slices tilted 25° towards coronal from the AC-PC line (2.3 mm isotropic) were collected covering the whole brain (TR = 0.65 s, TE = 34.8 ms, flip angle = 52°, matrix = 90×90, SMS factor = 8). Different slice prescriptions were used here for the different localizers to be consistent with the parameters used in our previous studies, and to optimize data collection for each paradigm (e.g., retinotopic mapping using a rotating checkerboard wedge benefits from a low TR to more finely capture phase-varying voxel responses). The slices were used to construct 3D brain volumes, which were then projected onto each participant’s cortical surface, thus placing the data from different localizers in a common anatomical space such that the exact slice prescriptions used had minimal impact on the final results.

### Data analysis

FMRI data were analyzed using FreeSurfer (surfer.nmr.mgh.harvard.edu), FsFast (A.M. Dale, Fischl, and Sereno, 1999) and in-house Python scripts. The exact same analysis pipeline was used for the two experiments, except that any analyses comparing clockwise versus counterclockwise spirals in Experiment 1 instead compared the spiky and curvy tessellation patterns in Experiment 2, due to the differing stimuli used. Preprocessing was performed using FsFast. All functional data was motion-corrected to the first image of the run of the experiment. Slicetiming correction was applied, but smoothing was not. A generalized linear model (GLM) with a boxcar function convolved with the canonical HRF was used to model the response of each trial, with the three motion parameters and a linear and quadratic trend used as covariates in the analysis. The first eight TRs of each run (prior to the presentation of the first stimulus) were included as nuisance regressors to remove them from further analysis. A beta value reflecting the brain response was extracted for each trial block in each voxel. ROIs were defined on the cortical surface (placing the results of the separate localizers in a common anatomical space) and then projected back to the native 3D functional space of the main experiment for further analysis.

#### ROI definitions

**Fig. 2.**
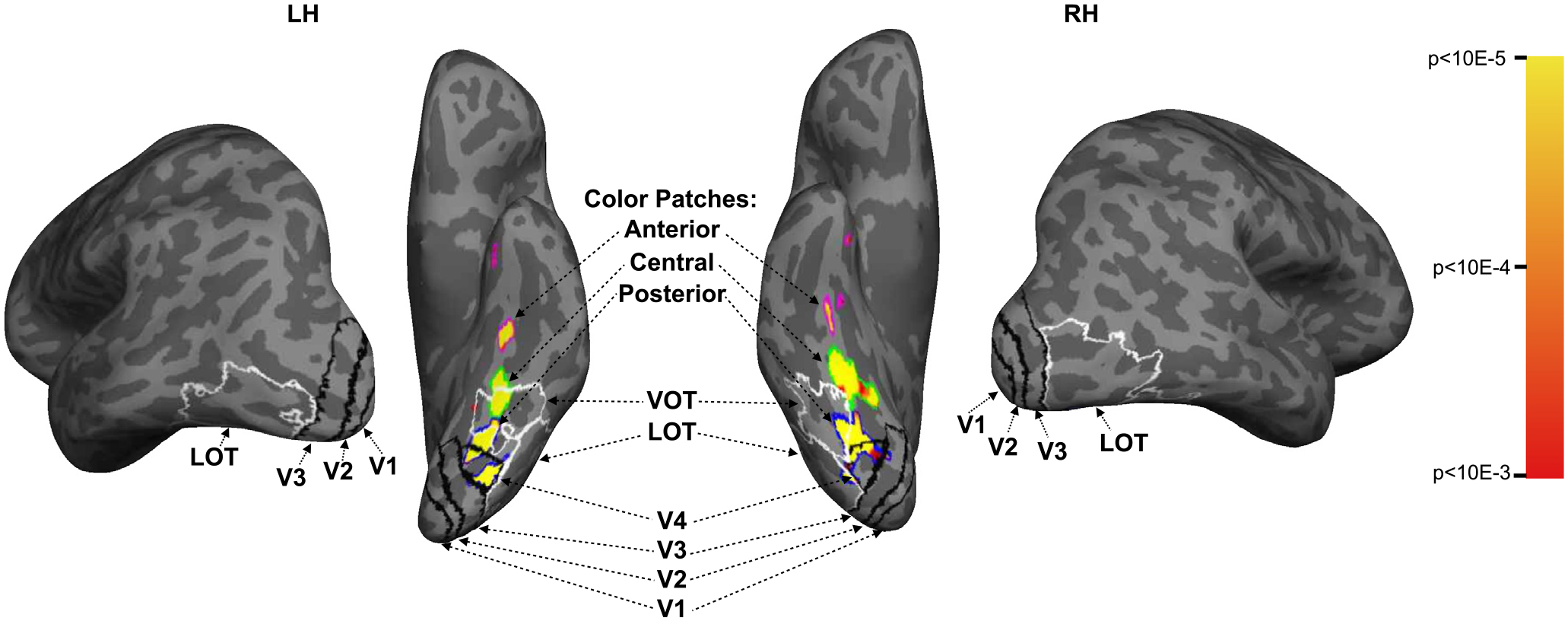
Lateral and ventral views of left and right hemispheres from an example participant, showing all regions of interest used in the study. Retinotopically defined areas V1, V2, V3, and V4 shown with black outlines; object-selective regions LOT and VOT shown with white outlines; posterior, central, and anterior color-selective regions shown with blue, green, and magenta outlines, respectively, along with their activation maps from the color versus greyscale localizer used to define them.

Using independent localizers, we defined ROIs in early visual areas and in higher visual regions showing univariate selectivity to shapes or colors. Fig. 2 depicts all ROIs for an example participant. For all ROIs, the results of the respective localizer paradigms described above were projected onto the cortical surface using Freesurfer and manually defined (details for different regions described below); ROIs were then converted to the native functional volume space of the main experiment to extract the voxels used in ROI analyses.

##### V1 to V4.

Areas V1 through V4 were localized on each participant’s cortical surface by manually tracing the borders of these visual maps activated by the vertical meridian of visual stimulation (identified by locating the phase reversals in the phase-encoded mapping), following the procedure outlined in Sereno et al. (1995).

##### LOT and VOT.

Following the procedure described by Kourtzi & Kanwisher (2001), LOT and VOT were defined as the clusters of voxels in lateral and ventral occipitotemporal cortex, respectively, that respond more to photos of real-world objects than to phase-scrambled versions of the same objects (*p* <. 001 uncorrected). These regions correspond to the location of LO and pFs (Malach et al., 1995; Grill-Spector et al., al.,1998; Kourtzi & Kanwisher, 2001), but extend further into the temporal cortex in our effort to include as many object-selective voxels as possible in occipito-temporal regions.

##### Ventral Stream Color Regions.

Following Lafer-Sousa et al. (2016), several color regions were identified in ventral temporal cortex as clusters of voxels responding more to colored images than to greyscale versions of the same images (*p* < .001, uncorrected). Since participants had varying numbers of such regions, we divided the regions in each hemisphere into anterior, central, and posterior color regions, following Lafer-Sousa et al. (2016). We were able to identify posterior and central color regions in every hemisphere of every participant in both experiments. In Experiment 1, we were able to localize the anterior color region in both hemispheres of 7/12 participants, one hemisphere of 3/12 participants, and neither hemisphere of 2/12 participants. In Experiment 2, we were able to localize the anterior color region in both hemispheres of 8/13 participants, one hemisphere of 3/13 participants, and neither hemisphere of 2/13 participants. The inconsistency in localizing this color region was possibly due to its location being close to the ear canals where large MRI susceptibility effects and signal dropoff could occur. We note that our rate of localizing this color region was similar to that of Lafer-Sousa et al. (2016), who reported that this region was found in both hemispheres of 6/13 participants, one hemisphere of 4/13 participants, and neither hemisphere of 3/13 participants. These anterior regions were generally relatively small (mean 49 voxels, std 46 voxels, min 4 voxels, max 163 voxels), precluding us from conducting meaningful decoding analyses in these regions. We thus omit them from further analysis. For reference, Supplemental Figure 1 shows these color-selective regions for all participants across both experiments (along with retinotopically-defined V4 for comparison), since fewer studies have examined these regions compared to the early visual and shape-selective regions.

##### V4 and VOT with Color Regions Removed.

We observed that the color regions overlapped with areas V4 and VOT in some cases. To document the extent to which color and form decoding in V4 and VOT might be affected by the color regions within them, we also ran several of the analyses on versions of V4 and VOT with the color-selective regions removed.

#### ROI overlap analysis

As noted just previously, we observed that areas V4 (defined retinotopically), the posterior color region (defined using a color versus greyscale localizer), and area VOT (defined using an object versus scrambled localizer) overlapped to some degree. To quantify this overlap, we computed the pairwise percent overlap between each of these ROIs, where percent overlap was defined as the percentage of the number of overlap voxels over the averaged number of voxels for the two ROIs, as we did in a previous study (Cant and Xu, 2012; see also Kung et al., 2007).

#### Multivoxel pattern analysis

In order to equate the number of voxels used in each ROI, the top 300 most active voxels in a stimulus-versus-rest GLM contrast across all the runs were selected. In addition to the ROIs described above, we also constructed an ROI for each participant consisting of the 300 most active voxels from the entire V1–V4 sector defined by the union of V1–V4, in order to test more sensitively for potentially subtle effects in several analyses. For several of the analyses (noted in each section below that describes the analysis), we analyzed subsets of the 100, 200, 300, 400, and 500 most active voxels per ROI, to determine the extent to which the presence of an effect depended on the number of voxels selected. A beta value was extracted from each voxel of each ROI for every trial block. To remove response amplitude differences across stimulus conditions, trial blocks and ROIs, beta values were z-normalized across all the voxels for each trial block in each ROI. For each of the contrasts of interest (described below), these beta values were used to train and test a linear support vector machine (SVM) classifier (with regularization parameter *c* = 1), using leave-one-run-out cross-validation. T-tests were performed to compare the decoding accuracy of the various measures to chance (one-sample, one-tailed *t*-test; one-tailed was used because below-chance decoding is not conceptually meaningful). To account for the fact that four participants partook in both experiments with the other participants being different between the two experiments, in cases where decoding was compared between pairs of conditions between the two experiments, a *partially-overlapping t-test* (Derrick et al., 2017) was performed. Likewise, to examine the influence of experiment, feature type, and their interaction on decoding in each region and between regions, a linear mixed effects analysis was performed (since this analysis, unlike the classical ANOVA, is able to explicitly account for subject-specific variance when only a subset of participants complete both experiments). Correction for multiple comparisons was applied using the Benjamini–Hochberg procedure with false discovery rate controlled at *q* < 0.05, with the details of this correction described for each analysis below (Benjamini and Hochberg, 1995). Specific details for each analysis were as follows.

##### Feature Decoding.

To assess the extent to which regions carried information about single features, in the single-conjunction blocks we trained and tested the classifier on color (red vs. green) and form (CW vs. CCW spirals in Experiment 1 and curvy vs. spiky tessellations in Experiment 2), where *both* values of the other feature were fed into each bin of the classifier (e.g., for color decoding, RedCW and RedCCW versus GreenCW and GreenCCW). Decoding for each condition was compared to chance (one sample *t*-test, one-tailed). Decoding for each feature was also compared between experiments (partially-overlapping *t*-test, two-tailed), and color and shape decoding compared within experiments (within-subjects *t*-test, two-tailed). Correction for multiple comparisons was performed within each ROI across analyses of the same kind: thus, for comparing decoding for each of the four conditions (two features by two experiments) to chance, correction for multiple comparisons was performed across these four comparisons; and for the four pairwise comparisons in each ROI (comparing color versus form decoding within each experiment, and comparing decoding for each feature across experiments), correction was performed across these four pairwise tests. We additionally performed mixed-effects analyses in each ROI to directly compare the results of the two experiments. The mixed-effect analysis is analogous to a two-way ANOVA, but takes into account the fact that a partially overlapping set of participants took part in the two experiments. We examined the main effect of feature type (color vs. form), the form features used in the two experiments (orientation and curvature), and their interaction. To test for broad trends in feature coding across the visual hierarchy, we also averaged the decoding accuracy of ROIs showing qualitatively similar response profiles via their proximity and their ordinal pattern of their feature decoding strengths over the two experiments, and the same analyses were performed for these sectors as were performed in the individual ROIs, with correction for multiple comparisons applied in the same manner. Note that this averaging of the individual ROI decoding accuracies across sectors is different from the V1–V4 macro-ROI described earlier that is used in other analyses, where decoding is performed once in a macro-ROI consisting of the most active voxels in the union of V1–V4. Further linear mixed-effects analyses were used to verify that the decoding profiles in these sectors in fact varied from one another.

Additionally, to document whether there exist any hemispheric differences in color and form coding, within each experiment we ran a within-subjects *t*-test between the left and right hemisphere for both color and form coding. Since this analysis was exploratory, no corrections for multiple comparisons were performed.

Finally, to examine the extent to which feature decoding results for V4 and VOT are driven by their overlap with the color regions, we constructed ROIs consisting of V4 and VOT minus their overlap with the color regions. The same feature decoding analyses were run for these ROIs as for the other ROIs, with the same correction for multiple comparisons applied. Additionally, two-way mixed-effects analyses with ROI and experiment as factors were run to examine whether decoding for either color or form significantly decreased in either region when the color-selective regions were removed (analyses conducted separately for each feature).

##### Feature Cross-Decoding.

To assess whether the two features were represented independently in each ROI (i.e., whether the representation of one feature was invariant to changes in the other feature) and whether there was any evidence of interactive feature coding, in the single conjunction blocks we performed cross-feature decoding in which we trained a classifier to discriminate two values of a relevant feature while the irrelevant feature was held at one value, and tested the classifier’s performance on the relevant feature when the irrelevant feature changed to the other value (e.g., train an orientation classifier on RedCW vs. RedCCW, and test orientation decoding on GreenCW vs. GreenCCW, or vice versa, with the results from the two directions averaged together). We did this for both features serving as the relevant feature. For comparison purposes, we also performed within-feature decoding, where we held the irrelevant feature constant between training and testing. This allowed us to compare the cross- and within-feature decoding using a matched number of trials. Decoding of each condition was compared to chance (one-sample *t*-test, one-tailed). Additionally, within-feature and cross-feature decoding were compared (within-subjects *t*-test, one-tailed; one-tailed was performed because only a decrease, not an increase, in performance from cross-decoding is interpretable) within each feature and experiment to determine whether coding for each feature is tolerant to changes in the other. Correction for multiple comparisons was performed within the set of comparisons done for each ROI (i.e., eight comparisons for comparing each condition to chance; four comparisons for comparing within-feature decoding to cross-decoding for the two experiments and two features).

Since both kinds of cross-decoding drop — a drop in color decoding across form features, or a drop in form decoding across colors — are conceptually similar in that they both reflect a more interactive feature representation, a one-tailed *t*-test was performed within each experiment to take both effects into account to test for an overall main effect of lower decoding in the cross-feature versus within-feature decoding conditions (note that this is the same as assessing a main effect of decoding difference between the within-feature and cross-feature decoding conditions across the two types of features using an ANOVA test, but looking at this main effect in a particular direction). Since this comparison provides critical evidence regarding whether or not interactive color and form coding may exist in a brain region, to perform an exhaustive search, we ran this particular analysis separately for the top 100, 200, 300, 400, and 500 most active voxels in each ROI, and also a combined V1–V4 ROI that includes the most active n voxels across the entire early visual sector. Given that SVM is sensitive to both power and noise (such that including too few voxels may exclude some of the informative voxels and thus provide insufficient power, whereas including too many voxels may add noise), testing the effect at a range of voxel sizes allowed us to assess the stability of any positive results obtained and how it may be affected by the number of voxels included in the analysis. Correction for multiple comparisons is applied within each voxel set, and separately within the early visual ROIs (since these constitute a replication of the results of Seymour et al., 2010) and the ventral stream ROIs; thus, correction is applied across five values for the early visual ROIs (V1, V2, V3, V4, and the combined V1–V4 macro-ROI; while the last is not strictly independent of the first four, we include it in the correction to err on the conservative side), and four values for the ventral stream ROIs (LOT, VOT, and the posterior and central color regions).

##### Pattern Difference MVPA.

**Fig. 3.**
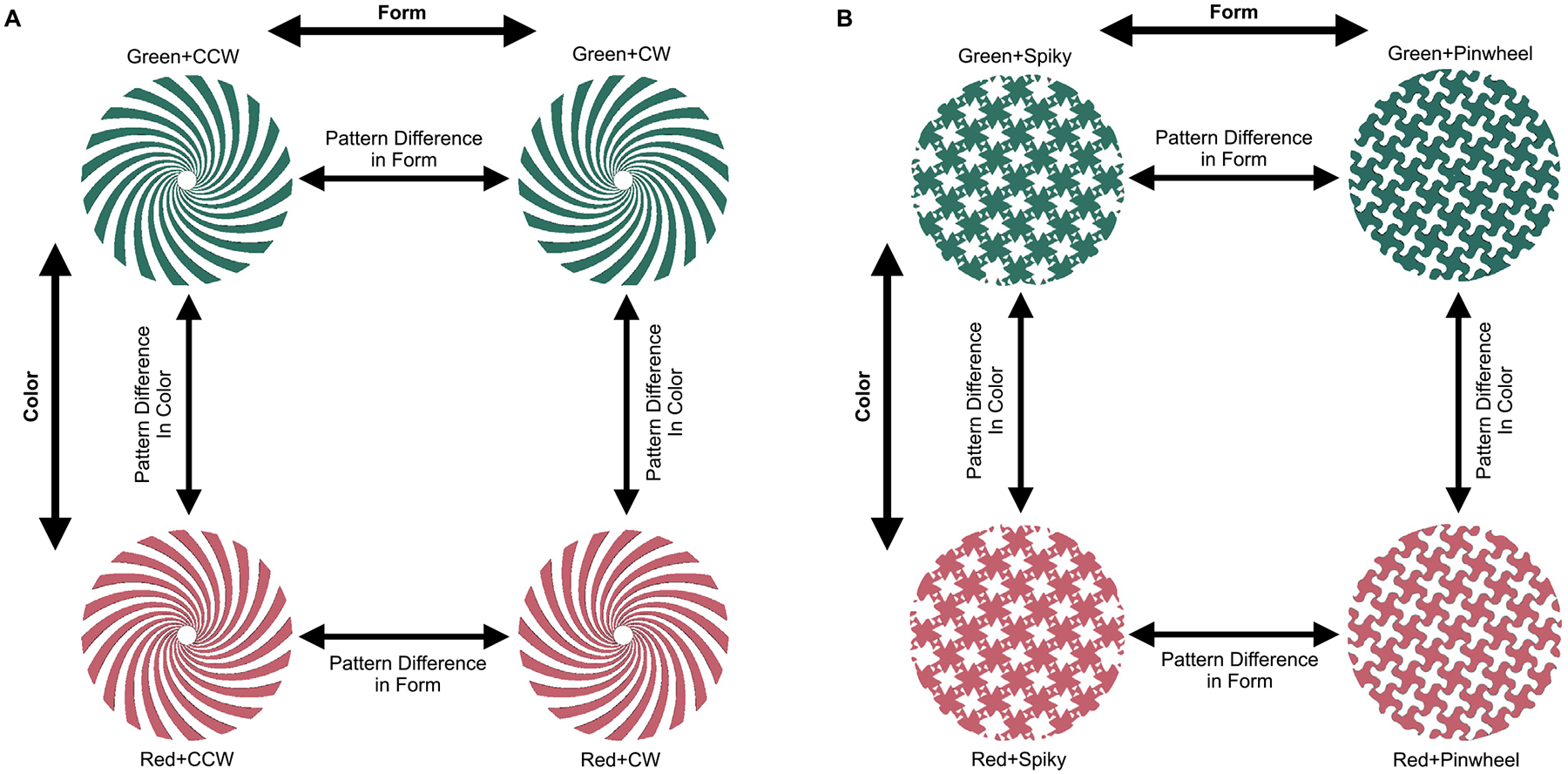
Logic of the pattern difference MVPA analysis for **A,** the color and orientation spiral stimuli in Experiment 1, and **B,** the color and curvature tessellation stimuli in Experiment 2. In this analysis, we examined which ROIs might code features in a manner that depends on the value of the other feature. From each ROI, we extracted and z-normalized the patterns associated with pairs of conditions matched on one feature but varying on the other, and took the difference between these patterns (e.g., GreenCCW - RedCCW). We did the same for the other value of the constant feature (e.g., GreenCW - RedCW). We then used SVM to determine whether these difference patterns were distinguishable from each other. This was done both possible ways — discriminating pattern differences in form across colors, and distinguishing pattern differences in color across the two values of each form feature — and the decoding accuracies were averaged.

To probe for the presence of interactive color and form representation in an ROI, we ran a novel analysis to examine whether the encoding of one feature (form or color) depends on the value of the other feature—that is, whether voxels in an ROI show aggregate evidence of a color-by-shape interaction effect in their tuning. Specifically, we first took the difference between the z-normalized beta values associated with RedCW and RedCCW, and between GreenCW and GreenCCW (Fig. 3). We then trained and tested an SVM (leave one run out cross-validation) on these difference vectors to examine whether the pattern differences between the two orientations change based on the color of the stimulus. We also performed the opposite analysis, comparing the beta value differences for the two different orientations (RedCW — GreenCW versus RedCCW — GreenCCW). The mean classification accuracies of these two directions of the analysis were then averaged, since an interaction effect implies a “difference of differences” in both directions (i.e., a difference in form pattern differences across colors, or a difference in color pattern differences across form features). Simulations with a known ground truth verified that the two directions of the analysis yield similar results, and so the results from the two directions were averaged rather than arbitrarily choosing one direction or the other. If the encoding of one feature is invariant to values of the other feature (i.e., the voxels exhibit only main effects with no interactions), SVM should discriminate these difference vectors at chance (50%); by contrast, if the encoding of one feature changes based on the other feature (i.e., an interaction effect), the classification should be above chance. Thus, the SVM classification step serves to aggregate small and potentially heterogeneous *interaction* effects across voxels (e.g., one voxel might show a superadditive interaction effect for RedCW, while another voxel might show a superadditive interaction effect for Green-CCW), analogous to how standard SVM decoding analyses aggregate small and potentially heterogeneous *pairwise* effects (e.g., some voxels might slightly prefer one condition and other voxels might prefer another) across voxels. The same analysis was performed for the tessellation stimuli in Experiment 2, replacing CW and CCW with the spiky and curvy stimulus conditions. One sample, one-tailed t-tests were performed for each ROI to determine if decoding of the pattern differences was above chance (one-tailed t-tests were used because below-chance decoding is not conceptually meaningful).

As in the cross-decoding drop analysis, we also ran this analysis separately on the top 100, 200, 300, 400, and 500 most active voxels in each ROI, so as to test exhaustively for the presence of interactive color-form coding in each ROI, and determine the extent to which the results depend upon the number of voxels selected; we also included the V1–V4 macro-ROI as in the previous analysis, and corrected for multiple comparisons in the same manner (separately within each voxel set and within the early visual and ventral stream ROIs).

We note that the information captured by this analysis is distinct from the information conveyed by feature cross-decoding. Feature cross-decoding would succeed so long as the pairs of patterns being cross-decoded end up on the correct side of the SVM decision boundary, even if the differences between the respective patterns were distinct (i.e., if main effects in feature coding far exceeded any interaction effects), or even if most units in the population exhibited interactive tuning, with just a small subset of units exhibiting strong invariant tuning for either feature (such that they provide an axis along which cross-decoding could succeed). By contrast, this method provides a more direct test regarding the existence of interactive coding in the representational space.

##### Double Conjunction Decoding.

As another way of examining which regions may contain interactive coding of color and form, we trained and tested the classifier on the two kinds of double conjunction blocks in each experiment (e.g., RedCW/GreenCCW and RedCCW/GreenCW). These blocks contained color and form features alternating once per second. Due to the sluggishness of the hemodynamic response, the pattern of BOLD activity present in each region would roughly constitute a superposition of the patterns associated with the two kinds of stimuli in each block. Since these two kinds of blocks both contained the two color and two form features used (e.g., red, green, clockwise, and counterclockwise), but differ in how they were conjoined, only regions encoding color and form in an interactive manner should be able to decode the two kinds of blocks from each other. The results of this analysis were compared against chance (50% decoding) using a one-sample, one-tailed *t*-test (one-tailed t-tests were used because below-chance decoding is not conceptually meaningful).

As in the cross-decoding drop and pattern difference analyses, we performed this analysis separately on the top 100, 200, 300, 400, and 500 most active voxels in each ROI and a V1–V4 macro-ROI consisting of the most active voxels across the entire sector. Correction for multiple comparisons is applied in the same way as the previous two analyses: within each voxel set, and separately within the early visual ROIs and the ventral stream ROIs.

## Results

Using fMRI MVPA, in the two experiments of this study, we examined the representation of simple and complex form features, color, and their conjunction in human early visual areas (V1 to V4) and higher-level ventral regions showing univariate selectivity to shape (LOT and VOT) and color (posterior and middle color regions) (see Fig. 2 for examples of these regions). This study served to both replicate the results of a study from Seymour et al. (2010), and extend their results from early visual cortex to higher-level ventral visual regions and from orientation to more complex form features. We aimed to understand the coding strength of these two types of features within a given brain region and across different brain regions along the ventral visual cortex, whether the multivariate feature selectivity of each region matches the univariate selectivity reported in past literature (e.g., Lafer-Sousa et al., 2016), and whether these two types of features are represented in a predominantly independent/orthogonal, or an interactive manner when representations of both features are found within the same brain region. We examined the coding of color and orientation in Experiment 1 by showing clockwise and counterclockwise spirals appearing in red and green colors, and the coding of color and curvature in Experiment 2 by showing spiky and curvy tessellations appearing in red and green colors. The phase of all stimuli alternated once per second, equating the overall stimulation across the visual field (and ruling out the possibility that any “form” decoding could merely be due to differences in the spatial envelope of the stimuli). In some of the runs, only a single stimulus type was present in each block. FMRI pattern decoding from these runs were used to determine which brain regions contain color and/or form information and how the relative coding strength of color and form may change across the ventral visual pathway. From these blocks, two analyses were used to test for the presence of independent versus interactive coding for color and shape: a standard cross-decoding analysis, and a novel method that explicitly tested for color-shape interaction effects in the voxel tuning across each ROI. In the other runs, these stimuli were presented in blocks where stimuli of different forms and colors were alternated, which we analyzed using a method adapted from Seymour et al. (2010) as another metric to test for the presence of interactive coding.

### ROI overlap

Since retinotopic V4, the posterior color region, and area VOT overlap to some degree, we quantified this overlap for each pair of these ROIs. Across all the participants from both Experiments 1 and 2, V4 and the posterior color region overlapped by 40.7% +/− 2.4% (mean +/− s.e.). VOT and the posterior color region overlapped by 16.4% +/− 2.7%. VOT and V4 overlapped by 17.5% +/− 3.5%. There is thus a sizable overlap between V4 and the posterior color region, with both also overlapping slightly with VOT. Despite these overlaps, as described below, there were significant differences in how color and form were represented in these brain regions that could not be predicted by the amount of anatomical overlap. Consequently, we grouped brain regions in a later analysis by their overall functional response profile, rather than by the amount of anatomical overlap.

### Color and form decoding

**Fig. 4.**
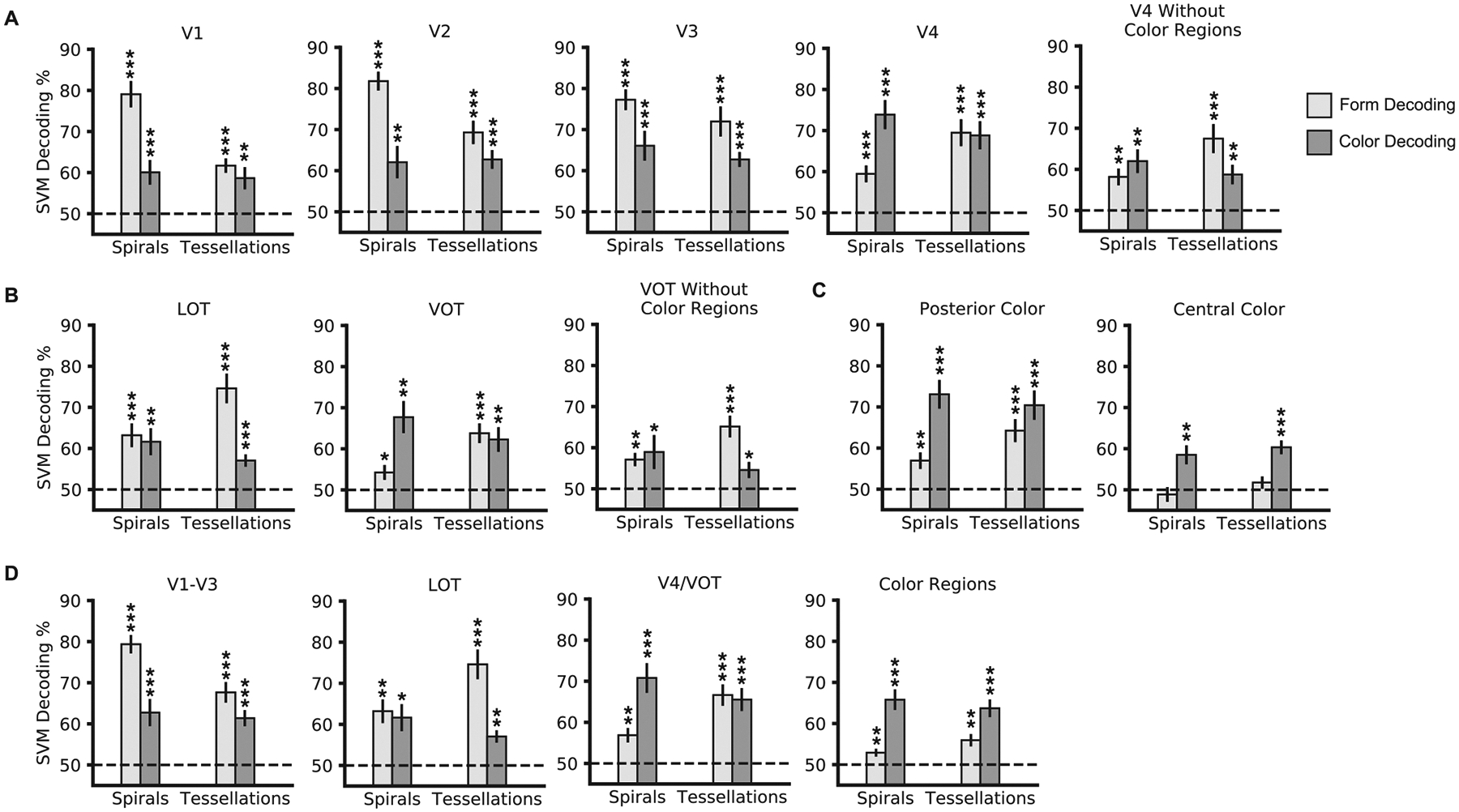
Results of color and form decoding in both experiments for **(A)** early visual areas, **(B)** shape regions, **(C)** color regions, and **(D)** sectors, which were formed by averaging the decoding of brain regions showing similar response profiles. Overall, V1 and V2 show a preference for orientation over curvature and color. V3 shows an equal preference to orientation and curvature over color. VOT and V4 showed equal preference to color and curvature over orientation; the overlap of V4 and VOT with the color regions partially, but not entirely, drove color decoding in these regions. Removing the color region overlap resulted in VOT showing a preference for curvature over orientation and color. LOT showed a preference for curvature over color and orientation. Lastly, the posterior color region showed a preference for color over orientation but not over curvature, while the central color region showed a preference for color over both form features. * *p* <0.05; ** *p* <0.01; *** *p* <0.001 for t-tests testing for above chance (> 50%) decoding (all one-sample t-tests, one-tailed, and corrected for multiple comparisons).

To document whether color and form information were present in a brain region, we compared color and form decoding accuracy in each region against chance level performance (Fig. 4). Here decoding was performed between fMRI response patterns differing in one feature dimension while allowing these patterns to take on either value of the other feature dimension (e.g., color decoding in Experiment 1 was performed by contrasting the red clockwise and red counterclockwise conditions against the green clockwise and green counterclockwise conditions). Except for the central color region, which showed no significant form decoding in either experiment (*ts* < 1.14, *ps* > 0.18), both color and form were decodable significantly above chance in both experiments in every brain region examined, including V1 through V4, the two shape regions LOT and VOT, and the posterior color region (*ts* > 2.27, *ps* < 0.03, one-tailed as only values above chance-level performance are meaningful here; results were corrected for multiple comparisons using the Benjamini-Hochberg procedure across the four tests within each ROI, since these tests were of the same kind; see Methods for more details). Fig. 4 depicts these results with the significance level of each *t*-test for above-chance decoding labeled with asterisks at the top of each bar. Color and form information is thus widely distributed throughout the ventral visual cortex, with both features present in every ROI tested with the exception of form information in the central color region.

To characterize the coding strength of color and the two types of form features (i.e., orientation and curvature) within a given region, we next conducted detailed comparisons within and across the two experiments (all statistical results are reported in Table 1). We noted that color coding did not vary between the two experiments in any of the brain regions examined even though only a subset of the participants completed both experiments. Because color stimulation was comparable between the two experiments (as the stimuli in both experiments subtended the same visual angle, with the same pixel-level presentation of colors), this suggests that participant performance at the group level was comparable and fairly stable across the two experiments. This enabled us to directly compare orientation and curvature coding between the two experiments and evaluate how the processing of these two form features may differ within a brain region. To account for the fact that partially overlapping sets of participants partook in both experiments, a linear mixed effects analysis (analogous to an ANOVA test) was performed to determine the influence of experiment, feature type, and their interaction on decoding in each region and between regions, and a partially-overlapping *t*-test (Derrick et al., 2017; analogous to a *t*-test) was performed to compare between pairs of conditions across the two experiments. Within each experiment, within-subjects t-tests were used to compare color and form decoding. For these pairwise t-tests (two comparing color and shape decoding within each experiment, and two comparing decoding for each feature across the two experiments), correction for multiple comparisons was applied across the four tests performed within each ROI.

As shown in Fig. 4 and Table 1, overall, in early visual areas, V1 and V2 showed a main effect of higher form than color decoding, with decoding further being higher for orientation than for either curvature or color. V3 also showed a main effect of higher form than color decoding, but with similar decoding for both form features. V4, on the other hand, showed a main effect of higher color than form decoding, with decoding further being higher for color and curvature than for orientation. In the two form-selective regions, VOT, like V4, showed a main effect of higher color than form decoding, with decoding further being higher for color and curvature than for orientation. LOT, on the other hand, showed no main effect of feature decoding, but higher decoding for curvature than for either color or orientation, consistent with its role in object shape processing. Both color regions showed a main effect of higher color than form decoding. While the posterior color region showed higher decoding for color and curvature than for orientation with no significant difference between decoding for color and curvature, the central color region showed higher decoding for color than for either kind of form feature.

Since V4 and VOT overlapped somewhat with the posterior color region, we performed additional analyses examining decoding in these regions when their overlap with the color-selective regions was removed. The same feature decoding analyses were performed in these regions as in the other regions (Fig. 4). Mixed-effects analyses were also performed for each feature across the two experiments to directly compare form and color decoding in these regions with or without the parts of these regions that overlapped with the color regions. For form decoding, V4 showed no main effect of overlap when the overlap with color-selective regions was removed (*Z* = 0.58, *p* = .57), but VOT showed a slight main effect with a trend towards an increase in form decoding (*Z* = 1.66, *p* = .096). However, for color decoding, in both ROIs there was a main effect of overlap, with color decoding significantly decreasing when the posterior color region was removed (*Z*s > 3.4, *p*s < 0.01), though color decoding remained significantly above chance (*ts* > 2.26, *ps* < 0.03, one-tailed; corrected for multiple comparisons across the four t-tests of decoding performance against chance in each ROI, as in all other ROIs). Removing the overlapping color region from V4 and VOT also changed the relative coding strength of color and form in these regions (see the detailed stats reported in Table 1). Both regions no longer showed an overall main effect of higher color than form decoding, with VOT now showing a greater sensitivity to curvature than to color or orientation changes. The latter is consistent with VOT’s role in object shape processing. Thus, removing the color-sensitive voxels from VOT and V4 removed their apparent feature preference for color.

Based on the overall similarity of their response profiles and their anatomical proximity, ROIs were grouped into sectors to allow us to directly compare the feature coding characteristics between the different sectors: early visual areas V1–V3, lateral visual area LOT, ventral visual areas V4/VOT, and Color Regions (including the posterior and central color regions). Decoding accuracies were averaged within each sector across the component brain regions. The decoding profiles within each sector are reported in Fig. 4 and Table 1, and they are overall consistent with the profile of the individual regions comprising the sector. Three-way mixed-effects models (sector × feature × experiment) performed on each pair of sectors reveal significant or trending two-way and/or three-way interactions involving sector for each pair, verifying that each of these sectors indeed exhibits a distinct feature encoding profile from each of the others (significant or trending effects included: for Color Regions vs. LOT, sector × feature and 3-way interaction; for Color Regions vs. V1–V3, sector × feature and 3-way interaction; for Color regions vs. V4/VOT, 3-way interaction; for LOT vs. V1–V3, sector × feature and 3-way interaction; for LOT vs. V4/VOT, sector × feature; for V1–V3 vs. V4/VOT, sector × feature and 3-way interaction; all *Zs* > 1.8, *ps* < 0.07).

We found only scattered and limited evidence for hemispheric differences in color or form coding. In Experiment 1, V1 showed higher form decoding in the right hemisphere, and V3 showed higher color decoding in the right hemisphere (*ts* > 2.36, *ps* < 0.05; both two-tailed and uncorrected), but these effects were not present in Experiment 2 (*ts* < *0*.60, *ps* > 0.56; two tailed and uncorrected), and no other ROIs exhibited a hemispheric difference for decoding of either feature (*ts* < 1.7, *ps* > *0*.12; two tailed and uncorrected).

Overall, with the exception of the central color region, all other regions examined showed significant decoding for both color and form, even for shape and color regions defined based on their univariate selectivity for color or form. At the same time, significant coding bias also exists in every region examined: even early visual areas show some feature coding preference, and in higher visual regions, such a preference appears to be largely consistent between multivariate decoding and the univariate feature preferences that define the regions.

### Color and form cross-decoding

**Fig. 5.**
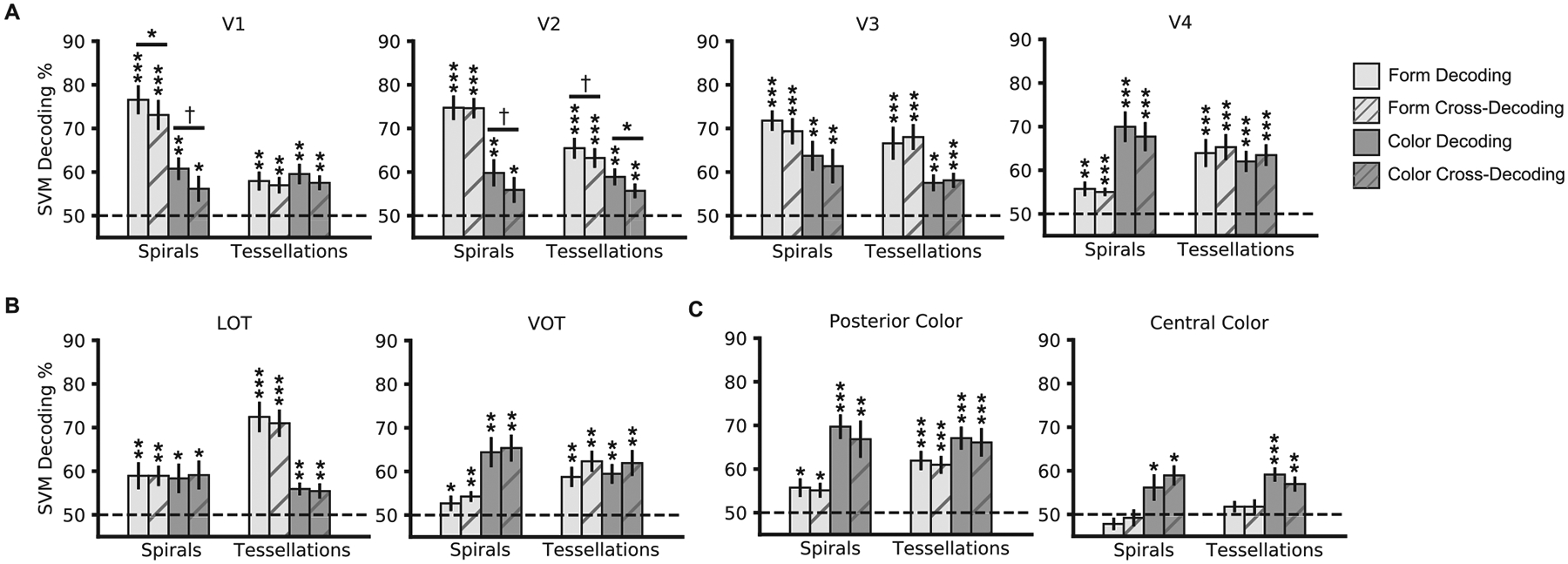
Results of feature cross-decoding analysis for **(A)** early visual areas, **(B)** shape regions, and **(C)** color regions. Solid bars show decoding accuracy for features trained and tested within the same value of the other feature (e.g., train on RedCW vs. RedCCW, test on RedCW vs. RedCCW); striped bars show decoding where training and testing for a feature is done across values of the other feature (e.g., train on RedCW vs. RedCCW, test on GreenCW vs. GreenCCW). Every region exhibiting successful decoding of a feature also exhibits significant cross-decoding; that said, V1 and V2 show a significant or trending drop in cross-decoding in several cases. † *p* <0.10, * *p* <0.05, ** *p* <0.01, *** *p* <0.001 for t-tests testing for above chance (> 50%) decoding (one-sample t-tests, one-tailed) and for t-tests testing for greater within-feature decoding than cross-decoding (within-subjects t-tests, one-tailed), all corrected for multiple comparisons.

To understand how color and form are coded together in a brain region, we next examined the extent to which each feature is encoded in a manner that is tolerant to changes in the other feature. To do so, we performed cross-decoding and trained an SVM classifier on one feature (e.g., form) within one value of the other feature (e.g., red), and tested the classifier in the other value of the other feature (e.g., green). Additionally, to obtain a baseline measure of feature decoding with an equal amount of data for comparison purposes, we also performed within-feature decoding, and trained and tested a classifier in one feature within the same value of the other feature. Fig. 5 depicts the results of these analyses. Every region that showed successful decoding of a given feature in the previous analysis also exhibited significant cross-decoding of that feature (*ts* > 1.92, *ps* < 0.05; one-tailed *t*-test, corrected for multiple comparisons with the eight comparisons performed for each ROI). Meanwhile, V1 and V2, but not other regions, also exhibited a significant or trending drop in decoding when performing cross-feature rather than within-feature decoding (Fig. 5): specifically, V1 showed a significant or trending cross-decoding drop for both color and orientation in Experiment 1 (*ts* > 2.00, *ps* < 0.08; one-tailed and corrected for the four cross-decoding tests within the ROI), and V2 exhibited a significant or trending cross-decoding drop for color in Experiment 1, and for both color and curvature in Experiment 2 (*ts* > 1.61, *ps* < 0.09; one-tailed and corrected for the four cross-decoding tests within the ROI).

**Fig. 6.**
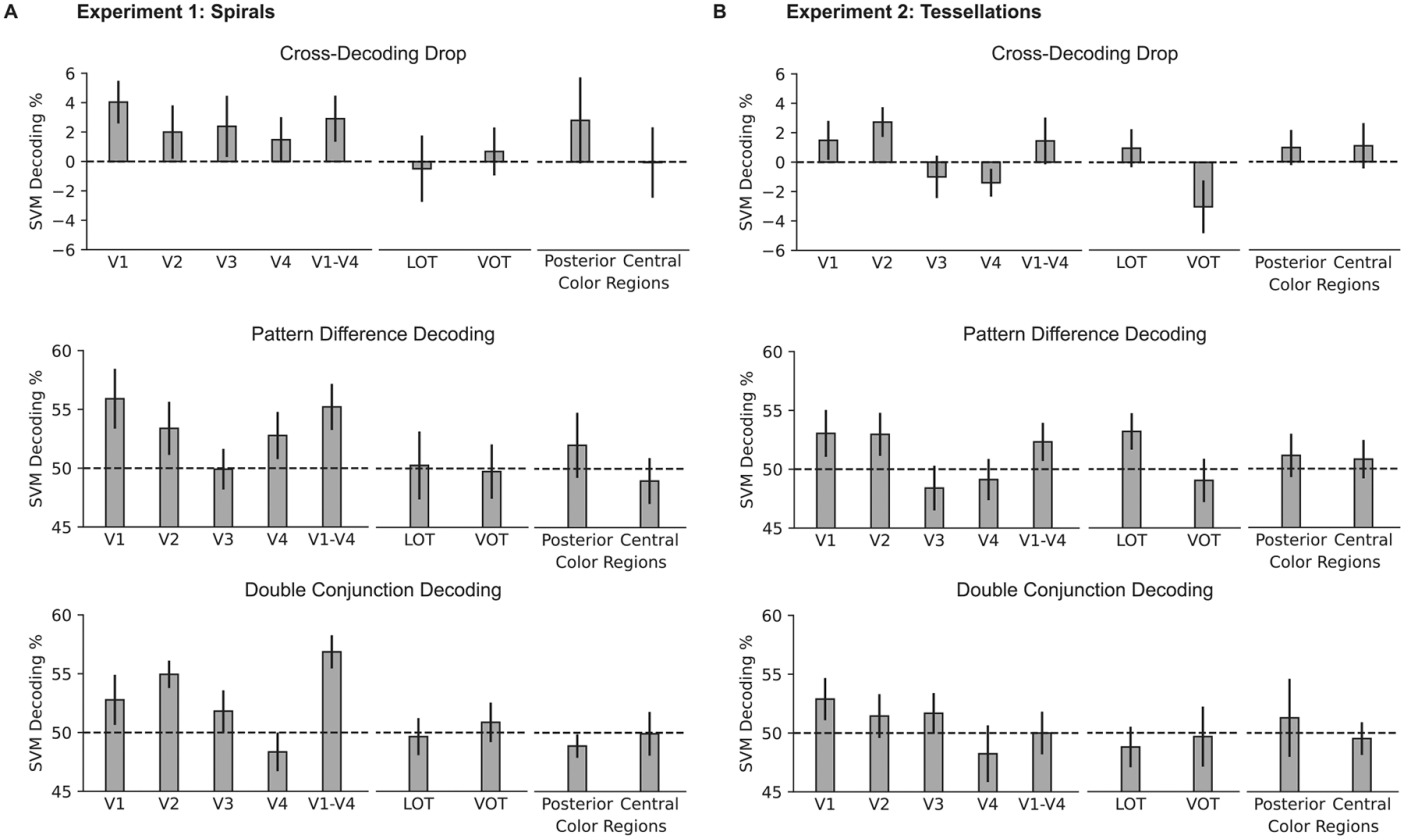
Results of the three analyses testing for interactive color/form coding — cross-decoding drop, pattern difference decoding, and double conjunction decoding — for Experiment 1 (A) and Experiment 2 (B), when the most active 300 voxels from each ROI were included in the analysis.

As the presence of a cross-decoding drop is an informative index of an interactive, rather than a completely orthogonal, relationship between color and form coding, to examine this effect in detail, we performed a set of further analyses. To increase power, we combined the effect from both color and form decoding (since a drop in either is suggestive of interactive coding between the features) and tested the amount of decoding drop in each ROI using one-tailed t-tests. Fig. 6 shows the results of this analysis for the main voxel set used throughout this study (i.e., 300 most active voxels in each ROI). To examine how the results may depend upon the number of voxels included in each ROI and reduce the possibility of obtaining null results due to too few or too many voxels being included, we also conducted this analysis separately for the top 100, 200, 300, 400, or 500 most active voxels in each ROI. Given that SVM is sensitive to both power and noise (such that including too few voxels may exclude some of the informative voxels and thus provide insufficient power whereas including too many voxels may add noise), testing the effect at a range of voxel sizes may provide us with a more sensitive way to document the effect. Tables 2 and 3 (top panel) show the results of this analysis for Experiment 1 (spirals) and Experiment 2 (tessellations), respectively. Correction for multiple comparisons was applied within each voxel set, and separately within the early visual and ventral ROIs.

In Experiment 1, V1, V2, and a macro-ROI composed of V1 through V4 exhibit a significant or trending drop in cross-decoding across multiple voxel selection conditions. By contrast, in Experiment 2, V2 showed a trend for a cross-decoding drop in just one voxel selection condition, and the posterior color region showed a significant cross-decoding drop in just one voxel selection condition. Thus, the strongest evidence for interactive coding based on the cross-decoding drop metric is for orientationcolor conjunctions in early visual regions. Other than these cases, however, color and form exhibit no significant drop in cross-decoding across the ventral visual pathway.

### Directly testing for interactive color-form coding using pattern difference analysis

Successful cross-decoding merely requires that the test patterns lie on the same side of the SVM classification boundary as the corresponding training patterns, and this can occur even in the presence of interactive tuning in the population. For example, a population with many units exhibiting interactive tuning can exhibit successful cross-decoding as long as the population also contains units with invariant tuning to the feature being cross-decoded, and so testing for a cross-decoding drop is only an indirect measurement of interactive tuning in a neural population. To remedy this and more directly test for interactive color-form coding across the human visual system, we performed a novel *pattern difference MVPA* analysis to specifically focus on the interactive effects that may be present in the response patterns in a brain region. Specifically, we extracted two *difference vectors*, each between two stimuli that differed on the same feature dimension (e.g., one difference vector could be RedCW minus GreenCW, while the other could be RedCCW minus GreenCCW). We then tested whether these two difference vectors could be discriminated using SVM. We did this separately for both color and form and then averaged the results (see Fig. 3 for a detailed illustration of this approach). If the encoding of one feature is completely independent and orthogonal to values of the other feature (i.e., only main effects), then chance-level decoding is expected; by contrast, if the encoding of one feature changes based on the other feature (i.e., an interaction effect), then above chance-level decoding is expected. This analysis essentially examines whether there is any interactive color and form coding in an ROI, with the SVM classification step serving to aggregate small interaction effects across voxels.

To test sensitively and exhaustively for the presence of interactive coding using this analysis method, and reduce the possibility that any null results are due to a nonoptimal number of voxels being used, for each ROI we performed the analysis separately for the top 100, 200, 300, 400, or 500 most active voxels. Fig. 6 depicts the results of this analysis for the top 300 most active voxels (since this was the main voxel set used throughout this study); Tables 2 and 3 (middle panel) show the results for all voxel sets. In Experiment 1 (spirals), we found trending or above-chance pattern difference decoding in multiple voxel sets from each of V1, V2, and the macro-ROI composed of V1–V4 (one-sample, one-tailed t-tests; corrected for multiple comparisons within each voxel set, and within each anatomical sector as described in Methods). In Experiment 2 (tessellations), we only found a trend in one voxel set each from V1 and the V1–V4 macro ROI, that did not replicate in any other voxel selection conditions. The overall pattern of results, then, is similar to that of the cross-decoding drop analysis: evidence of interactive color/form decoding is most reliably found in early visual cortex but not in higher-level ventral regions, and for orientation but not for curvature.

### Testing for interactive color-form coding using double conjunction decoding

As another way to test for the presence of interactive coding of color and form, in an independent set of data, following Seymour et al. (2010), we examined which ROIs are able to discriminate between two pairs of stimuli, where each pair has the same set of four individual features, but conjoined in different ways. Specifically, we trained a classifier to discriminate between two kinds of blocks, each consisting of alternating pairs of stimuli with different form and color features, such that the same set of four features is present in each kind of block, but combined in different ways (e.g., one kind of block alternated between RedCW spirals and GreenCCW spirals, and the other alternated between RedCCW and GreenCW spirals). If a region encodes these features in a completely additive, orthogonal manner, such that tuning to a feature does not depend on the value of the other feature, then patterns of activity in this region should not be able to distinguish these two kinds of block; by contrast, if there is any interactive coding of features, such that some voxels are sensitive to particular *pairings* of color and form features, then an SVM classifier should be able to distinguish these two kinds of blocks.

As in the decoding drop and pattern difference analyses, we performed this analysis separately on the top 100, 200, 300, 400, and 500 most active voxels from each ROI. Fig. 6 shows the results of this analysis for the set of 300 voxels, and Tables 2 and 3 (bottom panel) show the results for all voxel sets (one-sample, one-tailed *t*-test; correction for multiple comparisons within each voxel set, and within each anatomical sector as described in Methods). In Experiment 1 (spirals), we found above-chance or trending double conjunction decoding for multiple voxel sets in V1, V2, V3, and the V1–V4 macro ROI. A trend was found in V4 for just one voxel set. By contrast, in Experiment 2 (tessellations), a trend was found for one voxel set in the central color region, with no other trending or significant results in any voxel set or ROI.

In order to compare our results more directly with those of Seymour et al. (2010), we also re-ran the analysis with three changes to the pipeline to better match their analysis approach. First, we included all voxels falling under *p* < .01 in a task versus rest contrast, instead of using the top 300 voxels in such a contrast. Second, instead of z-normalizing the beta values going into the analysis across voxels within each trial, we normalized the beta values of each *voxel* across all its trials. Third, we did not apply correction for multiple comparisons. When we used the *p* < .01 activation threshold for voxel selection, we found no significant conjunction decoding in any individual ROI, or in the V1–V4 macro-ROI, with either within-voxel normalization (*ts* < 1.07, *ps* > 0.15, uncorrected) or across-voxel normalization (*ts* < 1.17, *ps* > 0.13, uncorrected), with the exception of a trend in V1 (*t(11)* = 1.56, *p* = .07, uncorrected). When we selected the most active 300 voxels (as we primarily used in our study), but used the within-voxel normalization method used by Seymour et al. (2010), we found significant conjunction coding in V2, V3, and the V1–V4 macro-ROI (*ts* > 2.62, *ps* < 0.02, uncorrected), along with a trend in V1 (*t(11)* = 1.59, *p* = .07, uncorrected) but no significant or trending decoding in V4 (*t(11)* = −0.14, *p* = .55, uncorrected). All in all, then, we replicate their finding of conjunction coding for V1, V2, and V3 when we apply the normalization method their study used, but not in V4.

## Discussion

Using fMRI pattern decoding and examining color and orientation coding in Experiment 1 and color and curvature coding in Experiment 2, the present study extends an earlier study by Seymour et al. (2010) and provides a comprehensive and updated documentation of the coding of color and form information across the ventral visual processing pathway in the human brain.

Broadly, we found that color and form information is nearly always anatomically commingled in the human ventral visual pathway. This includes early visual areas V1 to V4, thus replicating the color and form decoding results of Seymour et al. (2010), and previously documented higher ventral visual regions defined based on their univariate selectivity for color or shape, including the posterior color region, LOT, and VOT. This is especially striking in the case of LOT, since it is nowhere in the anatomical vicinity of the color-selective regions. The only exception to this pattern is the central color region which showed significant color decoding, but no form decoding, in both experiments, making it unique among the regions we examined. We were unable to reliably localize the anterior color region in every participant here due to its location near the MRI signal dropout zone (at a rate similar to Lafer-Sousa et al., 2016). Overall, across the human ventral visual processing pathway, we found a largely distributed representation of color and form features, even in higher visual regions defined by their univariate selectivity for one feature or the other.

That said, coding preference for either feature, quantified using MVPA, varied across regions, and depended on the specific form feature tested. V1 and V2 were most sensitive to orientation differences, and less so to either curvature and color differences, thus showing a preference for orientation over curvature and color. V3 showed higher sensitivity to either form feature than to color. VOT and V4, which greatly overlapped, showed equally strong sensitivity to color and curvature differences, but less sensitivity to orientation differences. The latter could potentially be due to the mirror symmetry of the clockwise and counterclockwise spirals used, since some evidence suggests that responses in VOT may be invariant to mirror-symmetric transformations (Dilks et al., 2011). The overlap of V4 and VOT with the color regions partially, but not entirely, drove color decoding in these regions: removing the color region overlap significantly decreased color decoding in these regions, but it remained above chance. Interestingly, removing the color region overlap also resulted in VOT showing a preference for curvature over orientation and color, consistent with this region’s univariate selectivity for complex object shapes. LOT showed roughly equal sensitivity to color and orientation changes, but far greater sensitivity to curvature changes, consistent with its univariate selectivity for complex object shapes. Finally, the posterior color region showed greater sensitivity to color than orientation, but an equal sensitivity to color and curvature, while the central color region showed a greater sensitivity to color than to either form feature. Thus, despite an overall distributed representation of color and form features, even early visual areas show a feature preference, and in higher visual regions, their feature preferences are largely consistent between the multivariate measures used in this study and their univariate feature selectivity extensively documented by previous studies. These results show that color and form features are represented in the human brain in a biased distributed manner.

That said, color and form information in different regions may potentially play different roles in visual information processing. For instance, achromatopsia patients can perceive isoluminant, color-defined shapes (e.g., a red square on a green background), even if they cannot report the colors that define the shape (Victor et al., 1989; Heywood et al., 1991; Barbur et al., 1994, 1998). This suggests that only feature information in some regions may be available to conscious perception.

To understand how color and form may be represented together in regions that code for both features, we examined the extent to which color and form are encoded in an *orthogonal* manner (with coding for each feature unaffected by the value of the other feature), an *interactive* manner (where coding for each feature depends on the value of the other feature), or some mixture of these motifs. In order to exhaustively test for the presence of interactive tuning and examine whether the results depend upon the set of voxels examined in each ROI, we performed each of these analyses on the 100, 200, 300, 400 and 500 most active voxels in each region.

Using a cross-decoding approach, we found most regions encode color and form information in a manner that is tolerant to changes in the other feature, demonstrating some independence in representation between these two features in each region. To assess the existence of interactive coding, we examined the amount of cross-decoding drop. We also devised a novel analysis method, *pattern difference MVPA*, that tests for the presence of multivariate interaction effects in voxel populations with greater sensitivity (see Methods). We reasoned that successful cross-decoding could coexist with interaction effects in a population when the interaction effects are small and leave the representations on the correct side of the classification boundary, or if interactively tuned voxels coexist with voxels that show strong independent tuning. By contrast, pattern difference MVPA presents a more direct test of interactive coding in a population. As a final test of interactive coding, in a separate data set, we also examine decoding using the double conjunction methodology developed by Seymour et al. (2010).

Across these three different analysis techniques and two independent datasets, we found evidence for interactive coding for color and orientation in early visual cortex, with these effects replicating across varying numbers of voxels included in the decoding analysis. These results largely aligned with those of Seymour et al. (2010), with one exception: while their study found significant interactive color/orientation coding in V4, we only found weak and non-replicable evidence for such coding in V4, only finding a trend for one analysis method in one voxel set. On the other hand, evidence for interactive coding for color and curvature was scarce, with no brain region showing replicable significant results across different analysis methods or voxel sets. Thus replicable evidence exists for interactive coding of color and form in early visual cortex and for simple form features, but not in higher-level visual regions or for more complex form features, where color and form appear to be encoded more orthogonally. It should be noted that even in early visual cortex we obtained much stronger decoding results for single features than for feature conjunctions and that cross-decoding accuracy was above chance. This suggests that, despite the presence of interactive color and orientation coding in early visual areas, color and form representations still exhibit a high degree of independence in all regions examined.

As an experimental method, fMRI depends on the heterogeneity of neuronal tuning across voxels at the probed spatial resolution. Our results thus should be understood within the limitations of this method, like all other fMRI studies. That said, the spatial scale measured by fMRI often reasonably tracks the documented spatial heterogeneity of neuronal feature tuning in several of the ROIs that were examined. For example, V1 orientation columns are organized at a scale visible to fMRI and plausibly contribute to fMRI MVPA decoding (Yacoub et al., 2008; Pratte et al., 2016), V2 neurons are organized into “stripe” patterns, approximately 1–2 m wide, with different kinds of stripes exhibiting different feature tuning (Ts’o et al., 2001), and monkey IT neurons are often organized into clusters 0.5 mm in diameter containing neurons with similar tuning (Wang et al., 1996; Tsunoda et al., 2001). As such, neural organization at the mesoscale visible to fMRI is not arbitrary or meaningless, but well-suited to capture the spatial tuning heterogeneity across neurons in many cases. This has enabled the representations visible to fMRI to be linked to the underlying neural computations, with fMRI decoding strength from human ventral and dorsal visual regions being tightly correlated with behavioral performance. For example, color decoding in V4, but not V1, reflected perceptual color space (Brouwer and Heeger, 2009), orientation decoding in early visual areas and superior intraparietal sulcus during the delay period of a visual working memory task tracked behavioral change detection performance (Bettencourt and Xu, 2016), and both object exemplar decoding and object category decoding in ventral and dorsal regions reflected perceived object similarity as measured by behavioral visual search and similarity judgement tasks (Mur et al., 2013; Charest et al., 2014; Jeong and Xu, 2016; Cohen et al., 2017; Xu and Vaziri-Pashkam, 2019). Thus, the mesoscale neuronal organization visible to fMRI can be used to probe the underlying neural computations.

In our study, decoding for each feature depends on the amount of variation we introduced within each feature. Because similarity within a feature likely changes across brain regions (e.g., two similar colors in one region may become dissimilar in another region), it would not have been possible to equate color and form variations for all the brain regions examined. Thus we have chosen what we believe to be reasonably large variations within each feature, including choosing two spirals with opposite directions, two tessellation stimuli with either all straight or all curved contours (thereby greatly varying an important midlevel form feature, curvature), and two hues that are maximally distinctive. These feature variations allow us to make a reasonable evaluation of the relative coding strength of color and form in each brain region, and more importantly, how the feature coding bias may change across visual regions. Although it could be argued that perhaps a wider array of colors and form features could have been sampled, by using a small number of stimuli chosen to greatly differ with respect to a chosen dimension (hue, orientation, curvature), we were able to maximize our power, giving us more confidence that any null results were not due to an inadequate number of trials. Furthermore, the logic of the double-conjunction design we used in one of the analyses requires two pairs of stimuli that differ with respect to two features.

One confound in comparing MVPA decoding across different experiments is that decoding accuracy can be affected not just by the strength of the underlying neural tuning, but also by factors like different analysis parameters, different levels of noise, and differences in data quality. For the present two experiments, however, the analysis pipelines were completely identical, removing analysis-related confounds. Furthermore, color decoding was statistically indistinguishable between experiments for every ROI, providing a common metric that suggests that levels of noise and data quality did not substantially differ, lending validity to the between-experiment comparisons. Another important confound in fMRI decoding approaches is that two experimental conditions can be discriminated by a linear classifier purely on the basis of differences in their noise covariance across voxels, even if their pattern centroids are the same (Hebart and Baker, 2018). Since our pattern difference analysis was novel, we therefore performed a control analysis in which we subtracted the mean pattern centroid of the training data within each condition to equate the pattern centroids between conditions while maintaining differences in covariance structure, and then fed these transformed patterns into our classifier. As a test case, we examined the macro-ROI consisting of the most active 300 voxels across V1–V4 in Experiment 1 (spirals), since we found replicable evidence for interactive color-form coding in this sector. We found chance level decoding with this data transformation (mean decoding accuracy 50.09%; *t(11)* = 0.21, *p* = .42 for one-sample, one-tailed *t*-test comparing against chance), suggesting that this confound does not account for our results.

One potential limitation of this study was that the stimuli were non-naturalistic and arguably “texture-like”. This may have contributed to several of the null results, such as the failure to find form coding in the central color patches (which Chang et al., 2017 found in the macaque), and the limited scope of conjunction coding that the study identified. However, one key advantage of the stimuli used was that, by repeatedly presenting the two complementary phases of the same stimuli we used in both experiments, it allowed the whole central visual field to be equally stimulated, increasing the odds of identifying conjunction decoding anywhere in the central visual field. Moreover, past work has found that object ensembles containing repeated shapes activate high-level object shape regions just like single objects, supporting the use of such stimuli to drive these regions (Cant and Xu, 2012). Although the stimuli in Experiment 2 were not scaled for eccentricity, this would only account for the null interactive coding findings if this coding motif only occurs over specific spatial scales, which would imply that it plays a rather specific rather than general role in visual processing.

In the present study, we found significant decoding of color and form much more reliably and broadly than we found evidence of interactive coding for these features, raising the question of what underlying patterns of neuronal tuning may account for these results. It is possible that neurons exhibiting interactive color/form tuning exist in higher-level ventral regions, but are not clustered in a sufficiently heterogeneous manner across voxels to be visible to fMRI MVPA. However, even if this is the case, it is interesting that such heterogeneity would be present for form- and color-coding neurons in higher-level ventral regions so as to enable decoding of individual features, and present for conjunction-coding neurons in early visual cortex so as to enable conjunction decoding in *these* regions, but absent for conjunction-coding neurons in higher-level ventral regions. At the very least, if these neuronal populations do exist, we can conclude that they are distributed very differently from the other neuronal populations involved in color and form coding in the ventral visual cortex. It is also possible such neuronal populations simply do not exist, thereby avoiding the potential combinatorial explosion involved in having dedicated neurons for encoding the combination of every form and every color.

Treisman and colleagues have famously argued that independently coded features can be conjoined via their shared location (Treisman and Gelade, 1980). One proposed neural mechanism for achieving this has been long-range synchronized firing between neurons corresponding to different features of the same object at the same spatial location (Singer, 1999), with the posterior parietal cortex (PPC) serving a critical role in mediating this process (Robertson, 2003) as damage to PPC can result in feature binding deficits (Cohen and Rafal, 1991; Friedman-Hill et al., 1995). However, it is unclear how such a code would be generated and read out, and the wiring patterns and temporal firing precision of neurons between brain regions may be insufficient to implement this code (Shadlen and Movshon, 1999). Nevertheless, binding through a shared location via a neural mechanism other than synchrony is still possible. Every region we examined was either defined through retinotopic mapping or plausibly overlaps with a region that exhibits retinotopy (e.g., the posterior color patches overlap with V4, and the central color patches potentially overlap with retinotopic regions VO1 and/or VO2; see Brewer et al., 2005; Larsson and Heeger, 2006; Wandell and Winawer, 2011). The co-existence of color and form representation, together with the presence of a detailed spatial map, could facilitate a binding by location mechanism at the local level without evoking long-range couplings between brain regions through neural synchrony, thereby serving as a potential binding mechanism (see also Di Lollo, 2012). How should we then bridge our results with the documented role of parietal cortex in binding? While past accounts posit that parietal cortex plays a purely spatial role in linking different features (e.g., Cohen and Rafal, 1991; Friedman-Hill et al., 1995), more recent accounts emphasize its role in the direct encoding and maintenance of task-relevant visual information (Bettencourt and Xu, 2016; Vaziri-Pashkam and Xu, 2017; Xu, 2017; Xu; 2018a, 2018b). It is possible that the commingling of color and form information on spatially organized ventral stream cortical maps serves to implicitly define the binding of features, but that parietal cortex must then explicitly extract these bindings for conscious perception and task-relevant processing. At minimum, the present study charts the anatomical layout and coding scheme of the ventral stream feature representations over which any putative parietal mechanism involved in feature binding might operate.

To conclude, our comprehensive approach illuminates the overall architecture of color and form processing in the human brain. Color and form information was not anatomically segregated into distinct anatomical regions defined by their univariate selectivity to either feature, but instead was generally co-localized in the same brain regions in a biased distributed manner throughout the ventral visual processing pathway, with decoding from color- and shape-selective regions largely consistent with their univariate preferences. Convergent evidence from three analyses and two independent data sets further shows that the joint coding of color and form within a region is overwhelmingly additive, with an additional (and relatively small) interactive component present in a subset of cases, reliably found only for the joint coding of color with simple form features in early visual cortex. Thus, the predominant relationship between color and form processing in the human ventral visual hierarchy appears to be one of anatomical coexistence but mostly representational independence.

## Supplementary Material

1 Supplemental Figure 1. Ventral view of brain showing color-sensitive patches for all participants; posterior color patches are shown in red, central color patches are shown in green, anterior color patches are shown in blue, and retinotopic V4 is shown as a black outline.

## Figures and Tables

**Table 1:** Summary of statistical comparisons within each ROI for color and form decoding results. Mixed-effects analyses were conducted to test the effect of experiment, feature type, and their interaction. Within-subject t-tests were conducted to test the difference between color and form decoding within each experiment. Partially-overlapping t-tests were conducted to compare the decoding of each feature across experiments.

	Main Effects and Interaction			Form vs. Color Within Experiment		Spirals vs. Tessellations Within Feature	
ROI	Experiment	Feature	Interaction	Spirals	Tessellations	Form	Color
**V1**	*z* = 1.06	*z* = 5.80	*z* = 3.51	*t(11)* = 4.81	*t(12)* = 1.10	*t(16.6)* = 6.21	*t(16.6)* = 0.97
	*p* = .29	*p* < .001	*p* < .001	*p* = .001	*p* = .34	*p* < .001	*p* = .34
		***	***	**		***	
**V2**	*z* = 0.029	*z* = 5.50	*z* = 2.56	*t(11)* = 5.13	*t(12)* = 2.05	*t(16.6)* = 4.12	*t(16.6)* = 0.20
	*p* = .98	*p* < .001	*p* = .01	*p* = .001	*p* = .09	*p* = .002	*p* = .84
		***	*	**	†	**	
**V3**	*z* = 0.85	*z* = 2.61	*z* = 0.082	*t(11)* = 2.21	*t(12)* = 2.71	*t(16.6)* = 1.14	*t(16.6)* = 1.20
	*p* = .39	*p* = .009	*p* = .93	*p* = .096	*p* = .075	*p* = .27	*p* = .27
		**		†	†		
**V4**	*z* = 0.74	*z* = 3.87	*z* = 2.91	*t(11)* = −3.88	*t(12)* = 0.18	*t(16.6)* = −2.59	*t(16.6)* = 1.17
	*p* = .46	*p* < .001	*p* = .004	*p* = .01	*p* = .85	*p* = .036	*p* = .34
		***	**	*		*	
**V4 w/out**	*z* = 0.69	*z* = 0.97	*z* = 2.31	*t(11)* = −0.15	*t(12)* = 2.01,	*t(16.6)* = −2.38	*t(16.6)* = 0.97
**Color**	*p* = .49	*p* = .33	*p* = .02	*p* = .34	*p* = .13	*p* = .11	*p* = .34
			*				
**LOT**	*z* = 1.11	*z* = 0.42	*z* = 3.1	*t(11)* = 0.40	*t(12)* = 4.93	*t(16.6)* = −2.52	*t(16.6)* = 1.24
	*p* = .26	*p* = .67	*p* = .002	*p* = .70	*p* = .001	*p* = .04	*p* = .31
			**		**	*	
**VOT**	*z* = 1.36	*z* = 4.06	*z* = 3.26	*t(11)* = −3.19	*t(12)* = 0.58	*t(16.6)* = −2.66	*t(16.6)* = 1.18
	*p* = .18	*p* < .001	*p* = .001	*p* = .03	*p* = .57	*p* = .033	*p* = .33
		***	**	*		*	
**VOT w/out**	*z* = 0.88	*z* = 0.55	*z* = 2.70	*t(11)* = −0.44	*t(12)* = 3.96,	*t(16.6)* = −2.59	*t(16.6)* = 0.92
**Color**	*p* = .37	*p* = .58	*p* = .007	*p* = 0.66	*p* = .008	*p* = 0.038	*p* = .49
			**		**	*	
**Posterior Color**	*z* = 0.62	*z* = 3.80	*z* = 1.69	*t(11)* = −3.33	*t(12)* = 1.59	*t(16.6)* = −2.23	*t(16.6)* = 0.61
	*p* = .54	*p* < .001	*p* = .091	*p* = 0.026	*p* = .18	*p* = .079	*p* = .55
		***	†	*		†	*p* = .55
**Central Color**	*z* = 0.72	*z* = 4.19	*z* = 0.33	*t(11)* = −3.27	*t(12)* = −5.71	*t(16.6)* = −1.44	*t(16.6)* = −0.65
	*p* = .47	*p* < .001	*p* = .74	*p* = 0.015	*p* < .001	*p* = 0.22	*p* = .52
		***		*	***		
**V1–V3**	*z* = 0.75	*z* = 5.51	*z* = 2.40	*t(11)* = 4.49,	*t(12)* = 2.64,	*t(16.6)* = 4.23	*t(16.6)* = 0.88
	*p* = .46	*p* < .001	*p* = .017	*p* = .002	*p* = .03	*p* = .002	*p* = .39
		***	*	**	*	**	
**V4/VOT**	*z* = 1.09	*z* = 4.50	*z* = 3.52	*t(11)* = −3.77	*t(12)* = 0.41	*t(16.6)* = −2.89	*t(16.6)* = 1.33
	*p* = .28	*p* < .001	*p* = .001	*p* = .012	*p* = .69	*p* = .02	*p* = .26
		***	**	*		*	
**Color**	*z* = 0.78	*z* = 5.33	*z* = 1.50	*t(11)* = −4.30	*t(12)* = −3.81	*t(16.6)* = −1.69	*t(16.6)* = 0.73
**Regions**	*p* = .43	*p* < .001	*p* = .13	*p* = .005	*p* = .005	*p* = .15	*p* = .47
		***		**	**		

† *p* < .10;

* *p* <0.05;

** *p* <0.01;

*** *p* <0.001 (all two-tailed, and corrected for multiple comparisons).

**Table 2:** Statistical results from Experiment 1 (spirals) for the three types of analyses that measure interactive coding for color and form: cross-decoding drop (top), pattern difference decoding (middle), and double conjunction decoding (bottom). Analyses were performed separately for the top 100 to 500 most active voxels in each ROI. All results were from one-sample, one-tailed t-tests examining whether the effects were significantly above chance. The first line of each cell shows the decoding accuracy (or cross-decoding accuracy drop) and standard error, and the second line shows the t-statistic and p-value separated by a slash; statistical significance is indicated with a marker on the right of each cell where applicable. Correction for multiple comparisons was applied across the set of ROIs within each combination of analysis, voxel set, and sector (e.g., across the 4 tests conducted for pattern difference decoding in the Top300 voxel set for the higher-level ventral stream sector).

ROI	Top100	Top200	Top300	Top400	Top500
	**Cross-Decoding Drop**				
**V1**	**2.5** (1.4) 1.78 / .085 †	**3.7** (1.6) 2.29 / .055 †	**4.0** (1.5) 2.67 / .055 †	**3.3** (1.6) 2.00 / .09 †	**2.5** (1.6) 1.48 / .14
**V2**	**3.6** (1.1) 3.18 / .02 *	**4.3** (1.4) 2.87 / .04 *	**2.0** (1.8) 1.05 / .19	**3.6** (2.2) 1.54 / .12	**2.2** (2.5) .85 / .26
**V3**	**0.5** (1.7) .31 / .48	**2.0** (1.5) 1.25 / .15	**2.4** (2.1) 1.10 / .19	**2.9** (1.9) 1.40 / .12	**4.1** (2.3) 1.73 / .14
**V4**	**−1.6** (1.8) −.82 / .78	**0.0** (1.5) 0.0 / .5	**1.5** (1.5) .92 / .19	**.9** (1.8) .46 / .33	**.8** (1.6) .49 / .32
**V1–V4**	**3.2** (1.1) 2.70 / .03 *	**1.5** (1.0) 1.48 / .14	**2.9** (1.6) 1.77 / .13	**3.6** (1.5) 2.24 / .09 †	**2.8** (1.5) 1.86 / .14
**LOT**	**−.3** (2.6) −.129 / .55	**−.5** (2.1) −.22 / .73	**−.5** (2.3) −.20 / .58	**0.0** (2.7) .016 / .67	**0.7** (2.2) .32 / .59
**VOT**	**−.3** (2.1) −.14 / .55	**.2** (2.1) .07 / .73	**.7** (1.6) .41 / .58	**0.0** (2.0) −.02 / .67	**−.4** (2.0) −.19 / .59
**Posterior Color**	**.4** (2.3) .18 / .55	**1.8** (2.8) .60 / .73	**2.8** (2.9) .92 / .58	**2.3** (2.5) .87 / .67	**2.5** (2.5) .94 / .59
**Central Color**	**.8** (2.2) .36 / .55	**−1.2** (1.9) −.62 / .73	**0.0** (2.4) −.02 / .58	**−1.3** (2.9) −.44 / .67	**−.6** (2.4) −.23 / .59
	**Pattern Difference Decoding**				
**V1**	**54.0** (2.4) 1.62 / .16	**55.4** (2.6) 2.01 / .09 †	**55.9** (2.5) 2.22 / .06 †	**54.6** (3.6) 1.23 / .15	**55.7** (2.6) 2.13 / .05 †
**V2**	**52.4** (1.7) 1.38 / .16	**56.8** (2.0) 3.28 / .02 *	**53.4** (2.3) 1.43 / .13	**55.5** (2.2) 2.37 / .047 *	**54.3** (2.0) 2.07 / .05 †
**V3**	**50.8** (1.3) .57 / .29	**51.3** (2.0) .62 / .27	**49.9** (1.7) −.05 / .51	**52.1** (1.6) 1.25 / .15	**51.9** (1.8) 1.01 / .21
**V4**	**51.9** (2.4) .75 / .29	**52.9** (2.4) 1.16 / .22	**52.8** (2.0) 1.33 / .13	**51.5** (2.2) .64 / .27	**51.7** (2.4) .70 / .25
**V1–V4**	**53.7** (2.5) 1.43 / .16	**51.4** (1.7) .76 / .27	**55.2** (2.0) 2.55 / .06 †	**56.1** (2.0) 2.84 / .04 *	**55.8** (2.0) 2.75 / .047 *
**LOT**	**48.2** (2.7) −.64 / .92	**50.9** (2.8) .30 / .76	**50.3** (2.9) .09 / .69	**50.3** (3.2) .10 / .82	**50.5** (2.9) .17 / .75
**VOT**	**47.2** (1.8) −1.50 / .92	**48.8** (2.2) −.52 / .89	**49.7** (2.3) −.11 / .69	**47.7** (2.4) −.94 / .82	**48.2** (2.5) −.70 / .75
**Posterior Color**	**51.5** (2.7) .53 / .92	**52.7** (2.7) .95 / .72	**52.0** (2.8) .69 / .69	**51.6** (2.8) .54 / .82	**50.3** (2.5) .13 / .75
**Central Color**	**49.1** (2.5) −.33 / .92	**47.7** (1.7) −1.3 / .89	**49.0** (1.9) −.51 / .69	**49.0** (2.3) −.39 / .82	**49.6** (2.2) −.19 / .75
	**Double Conjunction Decoding**				
**V1**	**55.9** (1.8) 3.06 / .03 *	**53.2** (1.9) 1.63 / .08 †	**52.8** (2.1) 1.25 / .20	**53.1** (1.6) 1.87 / .055 †	**52.4** (1.1) 2.10 / .049 *
**V2**	**51.9** (1.8) 1.02 / .21	**54.4** (1.4) 3.1 / .012 *	**54.9** (1.2) 4.07 / .002 **	**54.3** (1.1) 3.68 / .009 **	**54.3** (1.1) 3.79 / .008 **
**V3**	**51.0** (1.2) .82 / .21	**52.8** (1.6) 1.63 / .08 †	**51.8** (1.8) .99 / .21	**52.9** (1.4) 1.92 / .055 †	**52.4** (1.5) 1.55 / .09 †
**V4**	**52.2** (1.2) 1.80 / .08 †	**50.2** (1.1) .15 / .44	**48.4** (1.6) −.96 / .82	**50.1** (1.9) .044 / .48	**50.7** (2.1) .32 / .38
**V1–V4**	**53.2** (1.3) 2.39 / .045 *	**54.8** (1.1) 4.16 / .004 **	**56.9** (1.4) 4.64 / .002 **	**53.7** (1.5) 2.32 / .05 †	**55.6** (1.7) 3.17 / .01 *
**LOT**	**50.3** (1.5) .19 / .43	**50.0** (1.1) −.04 / .66	**49.7** (1.6) −.21 / .78	**50.2** (2.3) .07 / .77	**50.4** (1.8) .23 / .77
**VOT**	**51.6** (1.0) 1.59 / .28	**51.7** (1.6) 1.01 / .44	**50.9** (1.7) .50 / .78	**48.7** (1.6) −.76 / .77	**50.8** (1.6) .48 / .77
**Posterior Color**	**51.6** (1.5) 1.09 / .30	**51.3** (1.5) .80 / .44	**48.9** (1.0) −1.0 / .84	**49.8** (1.1) −.15 / .77	**49.3** (.8) −.83 / .79
**Central Color**	**51.2** (1.8) .62 / .37	**49.2** (1.8) −.42 / .66	**49.9** (1.9) −.05 / .78	**49** (2.3) −.41 / .77	**49.6** (2.2) −.19 / .77

† *p* < .10,

* *p* <0.05,

** *p* <0.01, and

*** *p* <0.001.

**Table 3:** Statistical results from Experiment 2 (tessellations) for the three types of analyses that measure interactive coding for color and form: cross-decoding drop (top), pattern difference decoding (middle), and double conjunction decoding (bottom). Analyses were performed separately for the top 100 to 500 most active voxels in each ROI. All results were from one-sample, one-tailed t-tests examining whether the effects were significantly above chance. The first line of each cell shows the decoding accuracy (or cross-decoding accuracy drop) and standard error, and the second line shows the t-statistic and p-value separated by a slash; statistical significance is indicated with a marker on the right of each cell where applicable. Correction for multiple comparisons was applied across the set of ROIs within each combination of analysis, voxel set, and sector (e.g., across the 4 tests conducted for pattern difference decoding in the Top300 voxel set for the higher-level ventral stream sector).

ROI	Top100	Top200	Top300	Top400	Top500
	**Cross-Decoding Drop**				
**V1**	**0.0** (1.2) −.03 / .51	**2.2** (1.3) 1.62 / .30	**1.5** (1.3) 1.08 / .33	**.9** (1.5) .58 / .47	**.8** (1.8) .42 / .57
**V2**	**1.8** (1.7) 1.04 / .51	**1.8** (1.4) 1.25 / .30	**2.7** (1.0) 2.59 / .06 †	**2.6** (1.5) 1.67 / .30	**1.3** (2.0) .64 / .57
**V3**	**.7** (1.5) .45 / .51	**−.5** (1.6) −.28 / .77	**−1.0** (1.4) −.67 / .91	**−1.7** (1.3) −1.29 / .89	**−1.5** (1.7) −.85 / .79
**V4**	**.2** (1.4) .14 / .51	**−1.8** (1.4) −1.21 / .88	**−1.4** (.9) −1.41 / .91	**−.4** (.8) −.48 / .85	**−.4** (1.0) −.39 / .79
**V1–V4**	**.3** (1.2) .23 / .51	**−.2** (1.3) −.17 / .77	**1.4** (1.6) .87 / .33	**1.2** (1.5) .78 / .47	**1.6** (1.6) .93 / .57
**LOT**	**−2.4** (1.3) −1.8 / .95	**1.0** (.6) 1.72 / .12	**1.0** (1.3) .71 / .34	**.8** (1.0) .78 / .51	**.1** (.9) .14 / .57
**VOT**	**−1.6** (1.3) −1.15 / .95	**−.9** (1.1) −.76 / .77	**−.3** (1.8) −1.6 / .93	**−1.5** (2.2) −.68 / .75	**−.2** (2.3) −.08 / .57
**Posterior Color**	**3.0** (1.0) 2.95 / .02 *	**2.0** (1.2) 1.67 / .12	**1.0** (1.2) .77 / .34	**.7** (1.0) .68 / .51	**−.2** (1.2) −.19 / .57
**Central Color**	**−1.6** (1.7) −.88 / .95	**.7** (1.3) .52 / .41	**1.1** (1.5) .67 / .34	**.3** (1.6) .17 / .58	**.4** (1.5) .28 / .57
	**Pattern Difference Decoding**				
**V1**	**50.7** (1.7) .40 / .57	**52.2** (2.1) 1.02 / .27	**53.0** (2.0) 1.47 / .16	**53.9** (1.5) 2.57 / .06 †	**52.2** (1.5) 1.44 / .22
**V2**	**52.3** (1.0) 2.19 / .12	**52.9** (1.6) 1.75 / .13	**53.0** (1.8) 1.56 / .16	**52.0** (2.0) .97 / .29	**52.6** (2.4) 1.08 / .25
**V3**	**50.2** (1.7) .09 / .57	**47.4** (1.5) −1.70 / .95	**48.4** (1.9) −.81 / .78	**47.9** (1.3) −1.53 / .92	**48.1** (1.6) −1.16 / .87
**V4**	**50.2** (2.1) .072 / .57	**50.4** (1.9) .21 / .53	**49.1** (1.8) −.48 / .78	**48.8** (1.7) −.69 / .92	**47.8** (1.8) −1.20 / .87
**V1–V4**	**49.7** (1.8) −.18 / .57	**52.6** (1.4) 1.78 / .13	**52.3** (1.6) 1.38 / .16	**54.3** (2.0) 2.10 / .07 †	**53.3** (1.8) 1.73 / .22
**LOT**	**49.9** (1.7) −.05 / .69	**50.6** (1.5) .40 / .35	**53.2** (1.6) 1.99 / .14	**50.4** (1.9) .20 / .56	**49.8** (2.3) −.07 / .59
**VOT**	**50.4** (1.9) .21 / .69	**50.6** (1.3) .41 / .35	**49.0** (1.8) −.50 / .69	**48.6** (2.3) −.58 / .71	**49.4** (2.2) −.24 / .59
**Posterior Color**	**51.8** (1.7) 1.00 / .67	**52.3** (1.6) 1.39 / .35	**51.1** (1.8) .59 / .43	**51.0** (1.7) .54 / .56	**49.5** (2.0) −.23 / .59
**Central Color**	**47.9** (1.4) −.14 / .91	**51.5** (1.7) .84 / .35	**50.8** (1.6) .47 / .43	**50.6** (1.4) .43 / .56	**49.7** (1.7) −.18 / .59
	**Double Conjunction Decoding**				
**V1**	**50.4** (1.3) .31 / .48	**52.3** (1.7) 1.34 / .17	**52.9** (1.8) 1.55 / .37	**51.6** (2.0) .79 / .43	**51.0** (2.0) .50 / .43
**V2**	**50.7** (1.6) .42 / .48	**52.6** (1.3) 1.86 / .17	**51.4** (1.9) .74 / .39	**51.4** (2.0) .66 / .43	**50.7** (1.7) .41 / .43
**V3**	**51.0** (1.9) .48 / .48	**49.2** (1.9) −.41 / .65	**51.7** (1.7) .94 / .39	**49.8** (1.4) −.11 / .59	**51.0** (2.3) .41 / .43
**V4**	**49.2** (1.6) −.49 / .68	**49.4** (1.9) −.33 / .63	**48.2** (2.4) −.70 / .75	**49.4** (2.3) −.24 / .59	**48.1** (2.5) −.73 / .76
**V1–V4**	**51.7** (1.8) .88 / .48	**51.9** (1.4) 1.3 / .17	**50.0** (1.8) 0.0 / .63	**51.8** (1.8) .97 / .43	**51.6** (1.6) .99 / .43
**LOT**	**49.4** (1.7) −.37 / .64	**49.9** (2.0) −.04 / .52	**48.8** (1.7) −.67 / .74	**51.1** (1.9) .58 / .49	**50.4** (2.2) .17 / .55
**VOT**	**50.7** (1.9) .36 / .53	**53.4** (3.0) 1.11 / .29	**49.7** (2.6) −.12 / .74	**49.5** (2.0) −.23 / .59	**49.8** (1.7) −.13 / .55
**Posterior Color**	**50.8** (3.0) .26 / .53	**50.7** (2.6) .27 / .52	**51.3** (3.3) .37 / .74	**51.2** (3.3) .35 / .49	**50.6** (3.1) .20 / .55
**Central Color**	**53.8** (1.6) 2.30 / .08 †	**51.8** (1.3) 1.31 / .29	**49.5** (1.4) −.33 / .74	**51.0** (1.3) .75 / .49	**50.8** (1.3) .60 / .55

† *p* < .10,

* *p* <0.05,

** *p* <0.01, and

*** *p* <0.001.

## Data Availability

We make our data (specifically, the beta values from each ROI for each run, subject and experiment) freely available via the Open Science Framework at https://osf.io/cma6p/. In conducting our analyses, we made use of several open source packages: Freesurfer’s FsFast pipeline (A.M. Dale, Fischl, and Sereno, 1999) for preprocessing the data and conducting GLMs, and various open source Python packages-specifically, Nilearn, Nibabel (https://github.com/nipy/nibabel/releases), and Scikit-Learn-for conducting all support vector machine analyses (Buitinck et al., 2013; Abraham et al., 2014).
