# Supplementary figures and images for: Representation of color, form, and their conjunction across the human ventral visual pathway
Source: Neuroimage. Author manuscript; Available in PMC 2022 May 1. (PMC9014861; doi:10.1016/j.neuroimage.2022.118941)

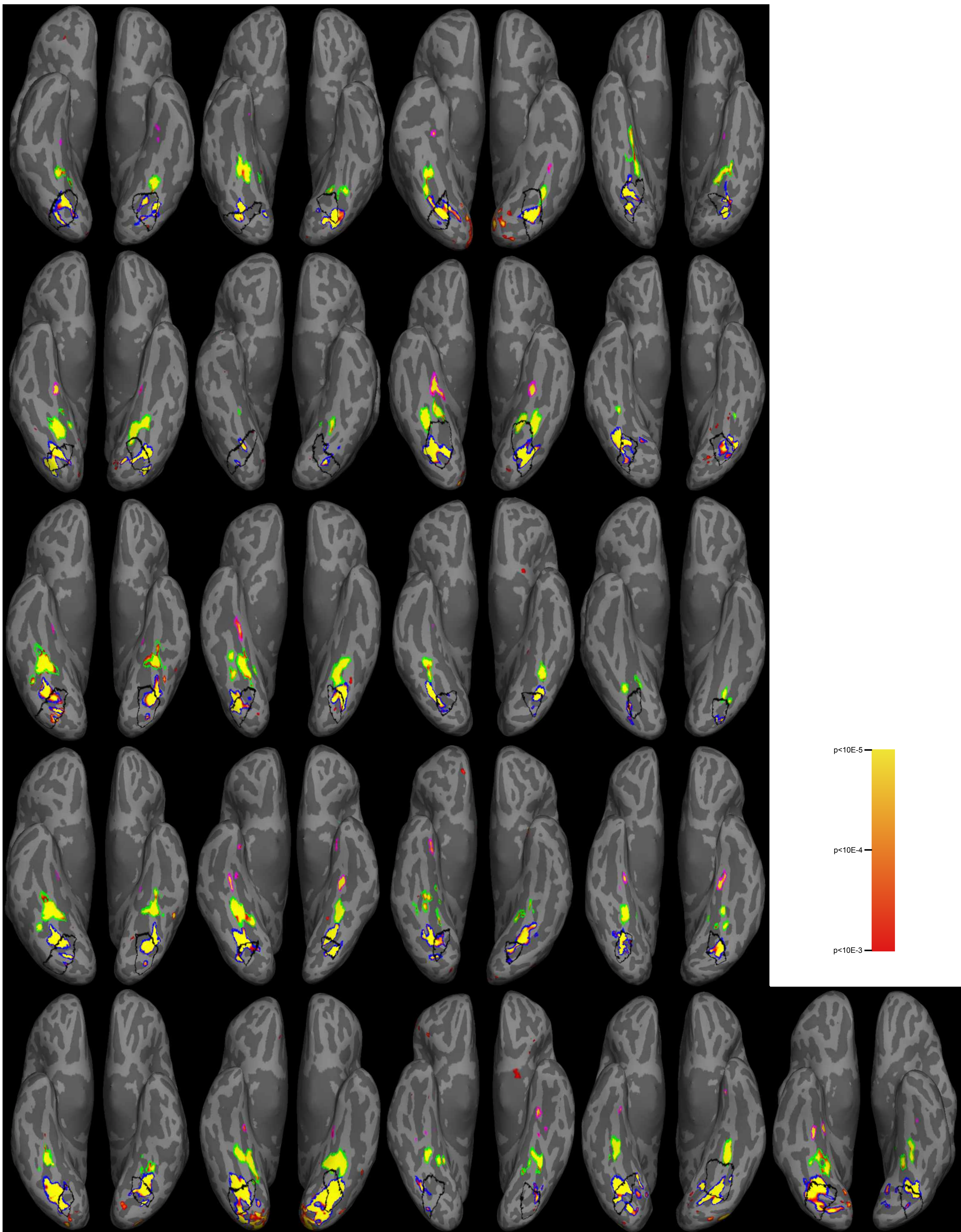

Supplement: 1 — Supplemental Figure 1. Ventral view of brain showing color-sensitive patches for all participants; posterior color patches are shown in red, central color patches are shown in green, anterior color patches are shown in blue, and retinotopic V4 is shown as a black outline. [file NIHMS1792869-supplement-1.pdf]
